# Regulation of ATR activity via the RNA polymerase II associated factors CDC73 and PNUTS-PP1

**DOI:** 10.1093/nar/gky1233

**Published:** 2018-12-12

**Authors:** Helga B Landsverk, Lise E Sandquist, Sreerama C Sridhara, Gro Elise Rødland, João C Sabino, Sérgio F de Almeida, Beata Grallert, Laura Trinkle-Mulcahy, Randi G Syljuåsen

**Affiliations:** 1 Department of Radiation Biology, Institute for Cancer Research, Norwegian Radium Hospital, Oslo University Hospital, Oslo, Norway; 2 Instituto de Medicina Molecular João Lobo Antunes, Faculdade de Medicina da Universidade de Lisboa, Lisboa, Portugal; 3 Department of Cellular and Molecular Medicine and Ottawa Institute of Systems Biology, University of Ottawa, Ottawa, Ontario, Canada

## Abstract

Ataxia telangiectasia mutated and Rad3-related (ATR) kinase is a key factor activated by DNA damage and replication stress. An alternative pathway for ATR activation has been proposed to occur via stalled RNA polymerase II (RNAPII). However, how RNAPII might signal to activate ATR remains unknown. Here, we show that ATR signaling is increased after depletion of the RNAPII phosphatase PNUTS-PP1, which dephosphorylates RNAPII in its carboxy-terminal domain (CTD). High ATR signaling was observed in the absence and presence of ionizing radiation, replication stress and even in G1, but did not correlate with DNA damage or RPA chromatin loading. R-loops were enhanced, but overexpression of EGFP-RNaseH1 only slightly reduced ATR signaling after PNUTS depletion. However, CDC73, which interacted with RNAPII in a phospho-CTD dependent manner, was required for the high ATR signaling, R-loop formation and for activation of the endogenous G2 checkpoint after depletion of PNUTS. In addition, ATR, RNAPII and CDC73 co-immunoprecipitated. Our results suggest a novel pathway involving RNAPII, CDC73 and PNUTS-PP1 in ATR signaling and give new insight into the diverse functions of ATR.

## INTRODUCTION

The ataxia telangiectasia mutated and Rad3-related (ATR) kinase is a master regulator of DNA-damage and replication-stress signaling coordinating DNA repair, cell cycle checkpoint and cell-death pathways (1). Understanding how ATR is activated is therefore a critical issue in biomedical research. The canonical pathway for ATR activation is initiated by the presence of single-stranded DNA (ssDNA) coated by RPA (ssDNA-RPA) (2). ssDNA-RPA at sites of DNA damage recruits ATR via its obligate binding partner ATRIP (2,3). Full activation of ATR is further facilitated by TOPBP1 (1). A large amount of evidence supports an important role for the canonical pathway in ATR activation (e.g. reviewed in (4)) However, there is also evidence suggesting the existence of alternative pathways (5), which are less well understood.

In one proposed alternative pathway the cell takes advantage of its transcription machinery to activate ATR (6,7). This was proposed based on the finding that upon stalling, elongating RNAPII could induce ATR-dependent P53 phosphorylation (7). RNAPII might thus act as a sensor for DNA damage (6). In fact, RNAPII is a recognised sensor in transcription-coupled repair where it recruits DNA-repair factors to sites of damage (8,9). The discovery of pervasive transcription outside protein coding genes (10), suggests that RNAPII might be scanning a majority of the genome and makes an involvement of RNAPII in sensing DNA damage and activating ATR conceivable (6). However, such an upstream role of RNAPII in ATR activation has yet to gain wide acceptance, perhaps because the factors involved in signaling between stalled RNAPII and ATR remain unknown.

During the transcription cycle, RNAPII becomes reversibly phosphorylated on the carboxy-terminal domain (CTD) of its largest subunit. Phosphorylation of specific residues in the CTD heptapeptide repeats, e.g. Ser 2 (S2) and Ser 5 (S5), is associated with specific phases of the transcription cycle. This is thought to contribute to a CTD ‘code’, in which combinations of post-translational modifications on the CTD can be ‘written’ and ‘read’ to regulate association with transcription and RNA processing factors (11). Interestingly, increased phosphorylation of the CTD has been observed after ultraviolet radiation and camptothecin in human cells (12,13) and is tightly connected to RNAPII stalling (14,15). Notably, RNAPII stalling can also occur after other types of stress, e.g. upon head-on collisions between RNAPII and the replication fork (16–18) or following ssDNA breaks or cyclopurines such as formed after IR (8,19–21). Furthermore, several proteins that interact with the phosphorylated CTD were required for resistance to ionizing radiation (IR) or doxorubicin in *Saccharomyces cerevisiae* (22). Based on these findings, one possibility would therefore be that RNAPII responds to stress by signaling via its CTD.

We previously discovered that siRNA-mediated depletion of the Protein Phosphatase 1 Nuclear Targeting Subunit (PNUTS) activates a G2 checkpoint in unperturbed cells and prolongs the G2 checkpoint after IR, but the underlying molecular mechanisms remained to be identified (23). Interestingly, PNUTS is one of the most abundant nuclear regulatory subunits of PP1 (24,25), and RNAPII CTD is the only identified substrate of PNUTS-PP1 (26). PNUTS-PP1 dephosphorylates RNAPII S5 (CTD) in vitro (27) and depletion of PNUTS causes enhanced RNAPII S5 phosphorylation (pRNAPII S5) in human cells (28). Because RNAPII, as described above, has a proposed role in ATR activation and ATR is a crucial player in the G2 checkpoint, we addressed whether PNUTS-PP1 might suppress ATR signaling. Our results show that ATR signaling increases after PNUTS depletion in a manner not simply correlating with DNA damage, R-loops or RPA chromatin loading. The increased ATR signaling rather appears to depend upon CTD phosphorylation, which is counteracted by PNUTS-PP1. Furthermore, the known phospho-CTD binding protein, CDC73, is required for the high ATR signaling, and ATR, RNAPII and CDC73 co-immunoprecipitates.

## MATERIALS AND METHODS

### Cell culture and treatments

Human cervical cancer HeLa and osteosarcoma U2OS cells were grown in Dulbecco's modified Eagle's medium (DMEM) containing 10% fetal calf serum (Life Technologies). The cell lines were authenticated by short tandem repeat profiling using Powerplex 16 (Promega) and regularly tested for mycoplasma contamination. HeLa BAC cells stably expressing EGFP mouse pnuts were a generous gift from the laboratory of Tony Hyman (http://hymanlab.mpi-cbg.de/bac_viewer/search.action). To generate the flag-CDC73 cell lines, CDC73 (Addgene plasmid # 11048) was amplified using the primers aggctttaaaggaaccaattcagtcgactgGAATTCGGATCCACCA (Cdc73 entry fwd) and aagaaagctgggtctagatatctcgagtgcTCAGAATCTCAAGTGCG (Cdc73 entry rev) and cloned into BamH1–Not1 cut pENTR1A using Gibson cloning (NEB E5510S). To generate the siRNA-resistant constructs, silent mutations were introduced in the siRNA target site using the Quick Change Lightning kit (Agilent 210518). The mutagenic primers were: CATCAGATGAAAAGAAGAAGCAGGGA-TGCCAGAGGGAAAATGAAACTCTAATACA and TGTATTAGAGTTTCATTTTCC-CTCTGGCATCCCTGCTTCTTCTTTTCATCTGATG. The construct was cloned into the lentiviral expression vector pCDH-eF1-GW-IRES-puro by Gateway cloning (Thermo-Fisher Scientific 11791020). HeLa cells were transduced and cells carrying the transgene were selected with 0.5 μg/ml puromycin.

Cells were irradiated in a Faxitron x-ray machine (160 kV, 6.3 mA, 1 Gy/min). Thymidine (Sigma-Aldrich) was used at 2 mM, Hydroxyurea was used at 80 μM, ATR-inhibitors VE-821 (Axon Medcem) and VE-822 (Selleck Biochem) at 10 and 1 μM respectively, CDK7-inhibitor THZ1 (ApexBio) at 1 μM, CDK9-inhibitor DRB (Sigma-Aldrich) at 100 μM, XPB-inhibitor triptolide (Sigma-Aldrich) at 1 μM and translational inhibitor cycloheximide (Sigma-Aldrich) at 10 μg/ml.

### siRNA and DNA transfections

Wildtype and RAXA (mutated in the ‘RVXF’ (^398^SVTW^401^) motif: V399A, W401A) full-length EGFP PNUTS DNA constructs containing 14 silent mutations in the domains targeted by siPNUTS (#1 and #2) were synthesized by Geneart and cloned into pGLAP3 (siPNUTS #2 is also called siPNUTS). pEGFP-RNaseH1 was a kind gift from Robert Crouch. Sequences of siRNA oligonucleotides can be found in supplementary Table S1. siRNA was transfected using Oligofectamine or RNAimax (Life technologies), and plasmid DNA with Fugene HD (Promega) or Attractene (Qiagen). Experiments were performed 65–72 h after siRNA transfection unless otherwise stated.

### Western blotting and antibodies

**Figure 1.**
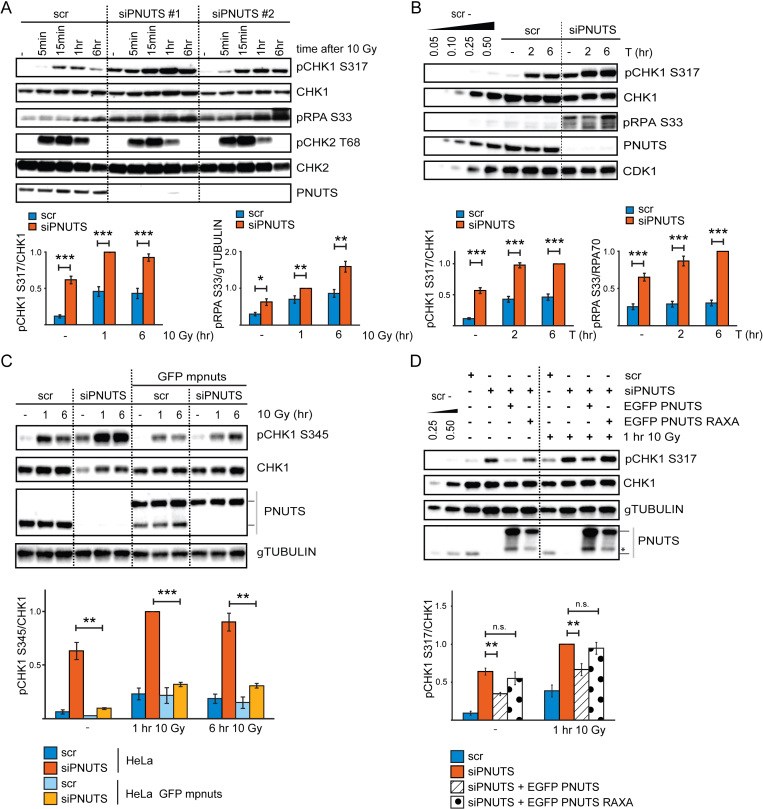
PNUTS-PP1 suppresses ATR signaling. (**A**) Western blot analysis of ATR and ATM signaling events in control scrambled siRNA transfected (scr) or PNUTS siRNA transfected (siPNUTS #1 and siPNUTS #2) HeLa cells, without IR or at indicated times after 10 Gy. Cells were harvested at 72 h after siRNA transfection. Bottom bar charts show quantification of pCHK1 S317 relative to CHK1 and pRPA S33 relative to γTUBULIN levels for siPNUTS #2, hereafter called siPNUTS (*n* = 8). (**B**) Western blot analysis of untreated cells or at 2 or 6 h after addition of thymidine to cells siRNA transfected as in A) (scr and siPNUTS). Bottom bar charts show quantification of pCHK1 S317 relative to CHK1 and pRPA S33 relative to RPA70 levels (*n* = 10). (**C**) Western blot analysis of HeLa cells or HeLa BAC clones stably expressing EGFP mouse pnuts (mpnuts) transfected with scr or siPNUTS (specifically targets human PNUTS), without IR or at 1 or 6 h after 10 Gy. Lines to the right of the western blot indicate migration of human endogenous PNUTS (lower band) and EGFP mpnuts (upper band). Bottom bar chart shows quantification of pCHK1 S345 relative to CHK1 levels (*n* = 3). (**D**) Western blot analysis of HeLa cells transfected with scr or siPNUTS. At 24 h post transfection, the indicated samples were transfected with wild type EGFP PNUTS or PP1-binding deficient EGFP PNUTS RAXA. Cells were harvested 48 h later without further treatment (–) or 1 h after 10 Gy. Lines to the right of the western blot indicate migration of endogenous PNUTS (lower band) and EGFP PNUTS/EGFP PNUTS RAXA (upper band), asterisk indicates what is likely EGFP PNUTS/EGFP PNUTS RAXA degradation products. Bar chart shows quantification of pCHK1 S317 relative to CHK1 (*n* = 3). Error bars indicate standard error of the mean (SEM) and statistical significance was calculated by the two-tailed Student's two sample t-test. *P < 0.05, **P < 0.01, ***P < 0.001

For quantitative western blotting, cells were resuspended in ice-cold TX-100 buffer (100 mM NaCl, 50 mM Tris pH 7.5, 2 mM MgCl_2_, 0.5% TX-100) containing 100 U/ml Benzonase (Sigma-Aldrich). After 1 h incubation on ice, Lane Marker Reducing Sample Buffer (Pierce Biotechnologies) was added and samples were boiled (95°C, 5 min). Criterion TGX gels (BioRad) and nitrocellulose membranes (BioRad) were used for separation and transfer respectively. Antibodies used are found in supplementary Table S2. Blots were imaged in a Chemidoc MP (BioRad) using chemiluminescence substrates (Supersignal west pico, dura or femto; Thermo Scientific). Quantifications were performed and images processed in Image Lab 4.1 (BioRad) software. Range of detection was verified by including a dilution series of one of the samples (see, e.g. Figure 1B) and excluding saturated signals. The resulting standard curve allowed accurate quantification. To blot for total protein after detection of a phosphorylated protein, membranes were stripped using ReBlot Plus Mild Antibody Stripping Solution (Millipore).

### Cell sorting and flow cytometry

For cell sorting and flow cytometry with EdU labeling, cells were labeled for 1 h with 2 μM EdU and fixed in 70% ethanol. EdU was labeled with the Click-iT Plus EdU Alexa Fluor 488 Flow Cytometry Assay Kit (Thermo Fisher), and DNA with FxCycle Far Red. Cells were sorted with a BD FACSAria Cell Sorter (BD Biosciences) using FlowJo software. Sorted cells were analyzed by western blotting as above. For flow cytometry analysis of RPA loading, we used a similar assay as one previously shown to detect end resection (29). Cells were pre-extracted, fixed and labeled as in (30) using anti-RPA70 antibodies (Cell Signaling). For flow cytometry analysis of γH2AX, samples were fixed and labeled as in (31). For simultaneous monitoring of EGFP-RNaseH1 with γH2AX and DNA, cells were fixed with 1% formalin in PBS for 1hr on ice, washed in PBS and resuspended in 70% ethanol. Samples were labeled with γH2AX antibody as in (30,31), but secondary antibody used was anti-mouse AlexaFluor568 (Thermo Fisher). In experiments in Figures 3E, F and 4C, barcoding of sets of four samples with pacific blue was performed as previously described (30) to eliminate variation in antibody staining between the individual samples. For analysis, a LSRII flow cytometer (BD Biosciences) was used with Diva or FlowJo software.

### Immunofluorescence

R-loops were detected as described previously (32). Briefly, U2OS cells were depleted for PNUTS and CDC73 using standard siRNA transfection for 72 h. siRNAs targeting the firefly luciferase were used as controls. After 72 h, cells were fixed and permeablized with 100% ice-cold methanol and acetone for 10 and 1 min on ice, respectively. Incubation with S9.6 antibody (ENH001, Kerafast) was followed by incubation with fluorochrome-conjugated antibodies Dy488 (Bethyl Laboratories). All the washing steps were done with PBS containing 0.05% (vol/vol) Tween 20. The intensity of the nucleoplasmic staining is plotted. At least, 50 cells from three independent experiments were scored.

For detection of RPA chromatin loading by immunofluorescence, HeLa cells were pre-extracted in detergent buffer (20 mM HEPES, pH 7.4; 50 mM NaCl; 1.5 mM MgCl_2_: 300 mM sucrose; 0.5% Triton X-100) for 5 min on ice prior to fixation with 4% paraformaldehyde. Cells were stained with anti-RPA32 in PBS-AT (PBS with 0.5% Triton X-100 and 1% BSA), followed by anti-mouse Alexa Fluor 568 (Thermo Fisher). All washing steps were done with PBS containing 0.01% (vol/vol) Tween 20. To stain DNA, cells were incubated briefly with Hoechst 33342. Mowiol (4-88, Sigma) was used for mounting. Cells were examined with a Zeiss LSM 710 confocal microscope (Carl Zeiss MicroImaging GmbH, Jena, Germany) equipped with an Ar-Laser Multiline (458/488/514 nm), a DPSS-561 10 (561 nm), a Laser diode 405–30 CW (405 nm), and a HeNe-laser (633 nm). The objective used was a Zeiss plan-Apochromat 63×NA/1.4 oil DICII. Image processing and analysis were performed with basic software ZEN 2011 (Carl Zeiss MicroImaging GmbH, Jena, Germany) and Imaris 7.7.2 (Bitplane AG, Zürich, Switzerland). Average intensity of RPA staining per nuclei (based on Hoechst 33342) was determined. In total, >130 cells for each condition from three independent experiments were analyzed.

### Immunoprecipitation experiments

For immunoprecipitations, cells were lyzed in TX-100 buffer (see under western blotting) containing 100 U/ml Benzonase (Sigma-Aldrich). Lysates were precleared and anti-CDC73 (Bethyl) or anti-pATR T1989 (GeneTex) or anti-RNAPII (F-12, Santa Cruz Biotechnologies) or anti-pCHK2 T68 (used as control antibody, from Cell Signaling) were added. Dynabeads (protein G; Life technologies) were used to isolate antibody-bound complexes.

### Statistics

All experiments, except when otherwise stated, were performed three times or more. Error bars represent standard error of mean (SEM). *P*-values were calculated with the two-tailed Student's one or two sample *t*-tests or the Mann–Whitney test.

## RESULTS

### PNUTS inhibits ATR signaling in a PP1-dependent manner

In our previous work (23), we observed increased phosphorylation of CHK1 and RPA32 at late timepoints (2-24 h) after IR in PNUTS depleted HeLa cells. As CHK1 and RPA32 are ATR targets (33,34), we addressed whether ATR signaling was affected specifically. Indeed, depletion of PNUTS with two different siRNA oligonucleotides caused increased IR-induced phosphorylation of the ATR substrates CHK1 S317 and RPA S33, but not of the ATM substrate CHK2 T68 (Figure 1A). Phosphorylation of CHK1 and RPA were increased both at early (5min-1h) and late (6h) timepoints after IR, as well as in the absence of IR (Figure 1A), suggesting a general role for PNUTS in suppressing ATR signaling. In agreement with this notion, pCHK1 S317 and pRPA S33 were higher also during thymidine-induced replication stress in PNUTS-depleted cells (Figure 1B). Similar results were found in U2OS cells (Supplementary Figure S1A), and the effect was clearly ATR-mediated, as the ATR inhibitor VE-821 inhibited the increased CHK1 phosphorylation after IR and thymidine (Supplementary Figure S1B,C). Inhibition of ATR activity was not a general effect after depletion of a PP1 regulatory subunit because knockdown of another abundant nuclear regulatory subunit, NIPP1 (24), did not increase CHK1 S317 or RPA S33 phosphorylation (Supplementary Figure S1D). Furthermore, the increased ATR signaling was not due to off-target effects of the siRNA oligonucleotides, since expression of mouse pnuts-EGFP to near endogenous levels abrogated the increased CHK1 phosphorylation after depletion of human PNUTS, both in the absence and presence of IR (Figure 1C).

To address the importance of PP1 for the inhibitory effects of PNUTS on ATR signaling, siRNA-resistant wild type and PP1-binding deficient PNUTS were overexpressed in cells depleted for endogenous PNUTS. Wild type PNUTS, but not the PNUTS-RAXA mutant deficient for PP1-binding (25), partially abrogated increased CHK1 phosphorylation in the absence of exogenous stress and after IR or thymidine (Figure 1D and Supplementary Figure S2A), showing that PP1-PNUTS binding is important for the negative effect of PNUTS on ATR signaling. Higher expression levels of the PNUTS RAXA mutant did not alter these results (Supplementary Figure S2B).

### ATR substrates CHK1 or RPA are not direct targets of PNUTS-PP1

**Figure 2.**
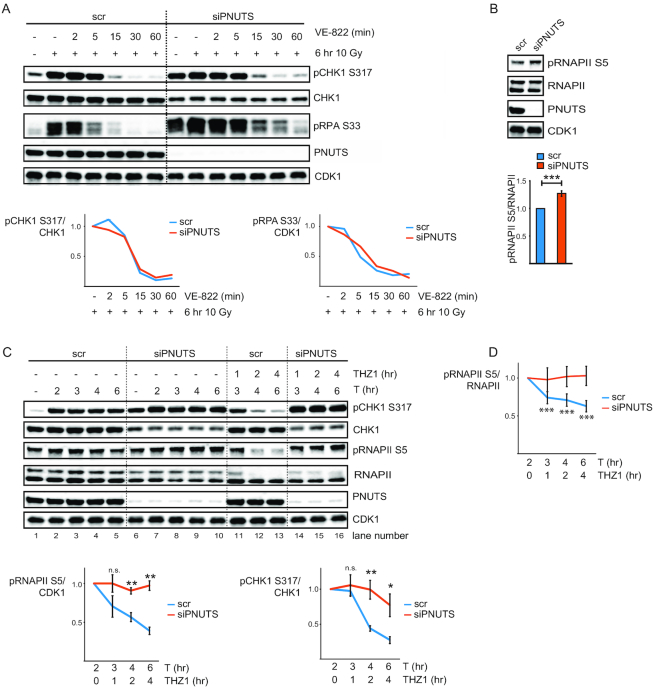
PNUTS-PP1 likely suppresses ATR signaling by dephosphorylating pRNAPII CTD. (**A**) Western blot analysis of scr or siPNUTS transfected cells without IR or 6 h after 10 Gy. VE-822 was added for 2, 5, 15, 30 or 60 min to indicated samples 6 h after 10 Gy. Charts show fold changes for VE-822-treated samples relative to the 10 Gy 6 h sample, for respective siRNA oligos from quantifications of pCHK1 S317 relative to CHK1 and pRPA S33 relative to CDK1. Experiment was performed 2 times with similar results. (**B**) Western blot analysis of scr and siPNUTS cells at 72 h after transfection. Bottom bar chart shows quantification of pRNAPII S5 relative to RNAPII (*n* = 14). ***P < 0.001 based on two-tailed Student's two sample *t*-test. (**C**) Western blot analysis of scr or siPNUTS transfected HeLa cells treated with thymidine for 2, 3, 4 and 6 h. THZ1 was added at 2 h after thymidine to the indicated samples. The bottom charts show fold changes for THZ1 and thymidine samples relative to the 2 h thymidine sample, for the respective siRNA oligonucleotides (*n* = 4) from quantifications of pRNAPII S5 relative to CDK1, and pCHK1 S317 relative to CHK1. Statistical significance was calculated from fold changes in scr versus siPNUTS samples at indicated timepoints by the two-tailed Student's two sample *t*-test. (**D**) Chart showing fold changes as in (C) from quantifications of pRNAPII S5 relative to RNAPII. Statistical significance was calculated with a two-tailed one sample t-test asking whether fold change after THZ1 was different from 1 (when the initial value prior to addition of THZ1 was set to 1) at the indicated timepoints for the respective siRNA oligonucleotides (*n* = 4). Note that the fold change after THZ1 in the siPNUTS transfected cells was not significantly different from 1 at any of the timepoints tested. *P < 0.05, **P < 0.01, ***P < 0.001. Error bars represent SEM.

Potentially, PNUTS-PP1 could counteract ATR signaling by generally dephosphorylating ATR substrates, as is the case for *Saccharomyces cerevisae* PP4 and the ATR homologue Mec1 (35). To address this, we added the ATR inhibitor VE-822 after induction of ATR signaling by IR. If PNUTS-PP1 directly dephosphorylates CHK1 and RPA, depletion of PNUTS should cause delayed removal of pCHK1 S317 and pRPA S33 after addition of the ATR inhibitor. However, both pCHK1 S317 and pRPA S33 declined at a similar rate in cells transfected with control siRNA and PNUTS siRNA (Figure 2A), showing that phosphatase activity against these substrates is similar under these conditions. Furthermore, overexpression of PNUTS did not decrease pCHK1 S317 or pRPA S33 relative to control transfected cells (Figure 1D and data not shown). These results strongly suggest PNUTS-PP1 does not directly dephosphorylate these ATR targets. To further verify this finding, we also examined pCHK1 S317/S345 and pRPA S33 after addition of the ATR inhibitor to thymidine-treated cells transfected with control siRNA and PNUTS siRNA (Supplementary Figure S2C). Decline of pCHK1 S317 and pCHK1 S345 occurred similarly also under these conditions, consistent with the notion that CHK1 is not a direct substrate of PNUTS-PP1. On the other hand, pRPA S33 declined less in PNUTS-depleted cells in the presence of thymidine (Supplementary Figure S2C). As pRPA S33 declined similarly in cells transfected with control and PNUTS siRNA after IR (Figure 2A), this most likely implies that another kinase contributes to pRPA S33 in PNUTS-depleted cells after prolonged replication stress (thymidine 16h). ATR-independent phosphorylation of pRPA S33 has e.g. been reported in the presence of hydroxyurea (HU) in combination with ATR inhibitor (36). Altogether, these results suggest that PNUTS-PP1 does not suppress ATR signaling by generally counteracting phosphorylation of its downstream substrates.

### Reduced dephosphorylation of RNAPII-CTD is likely promoting the high ATR signaling in cells depleted for PNUTS

As the RNAPII CTD is the only known direct substrate of PNUTS-PP1 (26,27), and RNAPII has a proposed role in ATR activation (6,7), we addressed whether dephosphorylation of RNAPII CTD is involved in the effects of PNUTS depletion on ATR signaling. We first verified that higher levels of pRNAPII S5 could be observed after depletion of PNUTS in HeLa cells (Figure 2B). We next added THZ1, a specific inhibitor of CDK7, the kinase mediating phosphorylation of RNAPII S5 (CTD) (37,38), to cells transfected with control siRNA or PNUTS siRNA during thymidine-induced replication stalling. To allow a robust activation of ATR signaling before inhibition of CDK7, thymidine was added 2 h prior to THZ1. Remarkably, both pRNAPII S5 and pCHK1 S317 were reduced upon addition of THZ1 to cells transfected with control siRNA (Figure 2C, lanes 11–13), and both pRNAPII S5 and pCHK1 S317 remained high in PNUTS-depleted cells (Figure 2C, lanes 14–16), suggesting that pCHK1 S317 depends on RNAPII CTD phosphorylation. Notably, the levels of pRNAPII S5 were reduced also when measured relative to total RNAPII after THZ1 in control siRNA transfected cells (Figure 2D). Also, while the ATR inhibitor VE-822 reduced pCHK1 S317 equally in cells depleted for PNUTS and cells transfected with control siRNA (Supplementary Figure S2C), the CDK7 inhibitor THZ1 only reduced pCHK1 S317 in cells transfected with control siRNA (Figure 2C), thus ruling out the possibility that THZ1 should directly inhibit ATR kinase.

The finding that pRNAPII S5 levels remained high in PNUTS-depleted cells after THZ1 treatment (Figure 2C) is consistent with a major role of PNUTS-PP1 in mediating the dephosphorylation of this residue (Figure 2C, compare lanes 14–16 with lanes 11–13). Moreover, depletion of another pRNAPII S5 phosphatase, SSU72 (39,40), also increased ATR signaling (Supplemental Figure S3A), supporting a role for pRNAPII S5 in ATR signaling. In addition, pRNAPII S2 and S7, two other phosphorylation sites on the RNAPII CTD also correlated with ATR signaling, as they were less reduced in PNUTS siRNA compared to control siRNA transfected cells after THZ1 (Supplemental Figure S3B). pRNAPII S2 and S7 may therefore also depend upon pRNAPII S5, and/or be direct targets of PNUTS-PP1. Interestingly, the effects of PNUTS-PP1 appeared to be most pronounced on pRNAPII S5, as pRNAPII S2 and S7 declined more than pRNAPII S5 after THZ1 in PNUTS siRNA treated cells, with average fold changes of 0.45 and 0.68 respectively, versus 0.97 at 4 h after THZ1 (Figure 2C and supplemental Figure S3B). Also, in contrast to pRNAPII S5 (Figure 2B) neither pRNAPII S2 nor S7 were significantly increased 72 h after PNUTS siRNA compared to control siRNA transfection (results not shown). Nevertheless, we cannot exclude a role for pRNAPII S2 and/or S7 in the high ATR signaling after depletion of PNUTS, and conclude that ATR signaling correlates with RNAPII CTD phosphorylation in general under these conditions.

To confirm the correlation between ATR signaling and RNAPII CTD phosphorylation, we added THZ1 to IR-treated cells. Similarly as observed during replication stress, pRNAPII S5 and pCHK1 S317/S345 were reduced after THZ1 in cells transfected with control siRNA (Supplementary Figure S3C, see charts and compare lanes 3–4 with 9–10). And again, pRNAPII S5 and pCHK1 S317/S345 remained higher in cells depleted for PNUTS (Supplementary Figure S3C, see charts and compare lanes 7–8 with 11–12). An inhibitor of translation, cycloheximide, did not reduce pRNAPII S5 and pCHK1 S317/S345 after IR neither in control nor in PNUTS-depleted cells (Supplementary Figure S3C, compare lanes 3–4 with 13–14 and lanes 7–8 with 15–16), suggesting the effects of THZ1 on ATR signaling are independent of *de novo* protein production (via transcription and translation). To further explore the correlation between RNAPII CTD phosphorylation and ATR signaling, THZ1 was added prior to IR. Consistent with a link between transcription and ATR, pCHK1 S317 was suppressed by THZ1 in HeLa cells (Supplementary Figure S3D). The effects of THZ1 on ATR signaling were likely mediated by RNAPII because similar effects were also obtained with 5,6-dichloro-1-β-d-ribofuranosylbenzimidazole (DRB) which inhibits transcription elongation via RNAPII (reviewed in (41,42)) and triptolide, which leads to the degradation of RNAPII (43). Notably, DRB and triptolide lead to reduced global levels of pRNAPII S5 (Supplementary Figure S3D). Also, translational inhibitor cycloheximide did not reduce pCHK1 S317 when added prior to IR (Supplementary Figure S3D). Collectively these results support a connection between RNAPII-driven transcription, RNAPII CTD phosphorylation and ATR signaling and suggest that PNUTS-PP1 inhibits ATR activity by dephosphorylating pRNAPII CTD.

### Enhanced ATR signaling occurs in G1 and in individual S-phase cells after depletion of PNUTS

**Figure 3.**
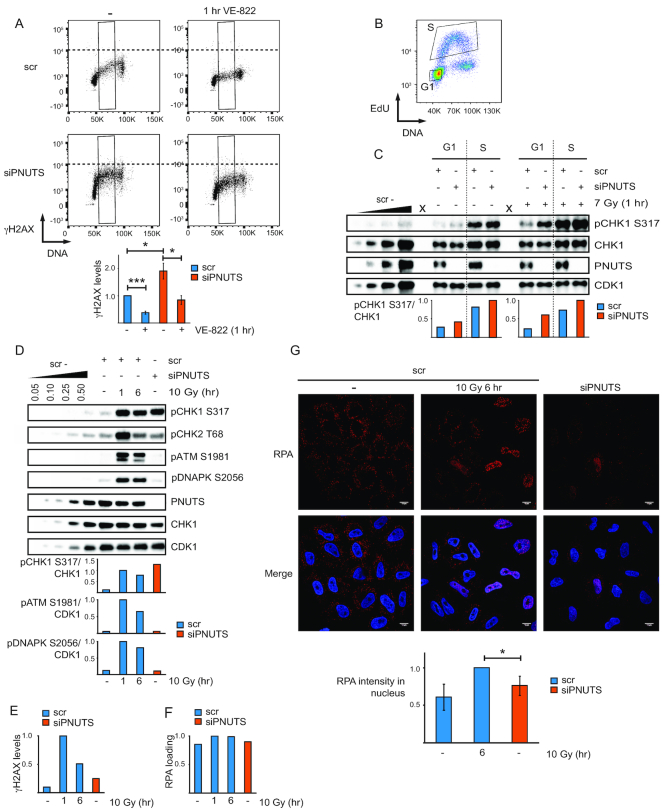
High ATR signaling after PNUTS depletion is present in individual cells, does not correlate with DNA damage markers and can occur in G1-phase. (**A**) Flow cytometry charts showing γH2AX versus DNA staining of individual scr and siPNUTS transfected cells with and without VE-822 for 1 h. S-phase cells were gated based on DNA content as indicated (black boxes). Quantifications show average median γH2AX levels in S-phase (*n* = 3). *P < 0.05, ***P < 0.001 based on two-tailed two sample Student's *t*-test. (**B**) Cell sorting was performed by flow cytometry into G1- and S-phases based on EdU incorporation and DNA content as indicated. (**C**) Western blot analysis and quantifications of sorted (as in B) scr and siPNUTS transfected HeLa cells. Cells were harvested at 48 h after siRNA transfection, with and without IR (harvested at 1 h after 7 Gy). Irradiation was performed immediately prior to addition of EdU. One representative image is shown, with X indicating empty lanes. Quantifications were performed on images with different exposure times for the non-irradiated and irradiated samples (due to their different intensities), and normalized to the respective siPNUTS S-phase sample. The experiment was performed three times, two at 72 h and one at 48 h after siRNA transfection with similar results. (**D**) Western blot analysis of DNA damage markers for scr (without IR or 1 and 6 h after 10 Gy) and siPNUTS transfected cells 48 h after siRNA transfection. (**E**) Bar chart showing median levels of γH2AX from flow cytometry analysis from cells harvested in parallel with samples from the same experiment in D. The samples were barcoded with pacific blue and mixed prior to staining to minimise sample to sample variation. The experiment in (E) compared to (D) was performed two times with similar conditions and results. (**F**) Bar chart showing median levels of RPA loading from flow cytometry analysis of pre-extracted cells from the same experiment as in (D). Samples were barcoded as in (E). The experiment in (F) compared to (D) was performed three times with similar conditions and results. (**G**) Immunofluorescence analysis of pre-extracted cells treated as in (D), but harvested at 72 h after siRNA transfection. Bottom bar chart shows average intensity of nuclear RPA staining from three independent experiments. *P < 0.05 for using two-tailed one sample Student's *t*-test (to test if RPA values in siPNUTS sample was different than 1, which we had set scr 10 Gy 6 h sample to). >130 cells were scored per condition in total. Error bars represent SEM.

ATR plays a major role in regulation of DNA replication and is known to be active in S-phase even in the absence of exogenous stress (reviewed in (44)). Potentially, high ATR signaling might therefore simply reflect a larger number of S-phase cells. As γH2AX in S-phase is ATR-dependent (45), we addressed this issue by simultaneously assessing γH2AX levels and cell-cycle position in individual cells after transfection with PNUTS siRNA- or control siRNA. ATR-dependent γH2AX levels in individual S-phase cells were higher after PNUTS depletion (Figure 3A). Therefore, higher ATR signaling following depletion of PNUTS cannot simply be explained by more cells in S-phase.

On the other hand, an accumulation of cells in S-phase could be observed after transfection with PNUTS siRNA (Supplementary Figure S4A), indicating effects on replication. We therefore compared ATR signaling after PNUTS depletion with the ATR signaling resulting from treatment with hydroxyurea (HU), a drug that is thought to activate ATR primarily by causing replication stress. HeLa cells treated with 80 μM HU for 24 h showed similar levels of replication stalling and percentage of cells in S-phase compared to PNUTS-depleted cells 48 h after siRNA transfection, as measured by uptake of the nucleoside analog EdU (Supplementary Figure S4B and S4E). However, pCHK1 S317 and S345 were clearly higher in the PNUTS depleted cells (Supplementary Figure S4C and D), strongly suggesting that the high ATR activity after depletion of PNUTS is not caused by replication stress alone.

Interestingly, previous studies have suggested that blockage of elongating RNAPII is sufficient to induce ATR signaling in human cells (7), and ATR has been shown to be activated in G1-phase (46,47), when replication does not occur. We reasoned that signaling via phosphorylated RNAPII CTD might be a mechanism permitting ATR activation in G1. To address this issue, cells in G1- and S-phases of the cell cycle were sorted based on EdU incorporation and DNA content (Figure 3B). Remarkably, pCHK1 S317 was higher in both G1- and S-phase after depletion of PNUTS, with and without IR (Figure 3C). To validate the purity of the G1-population following sorting, thymidine, which specifically targets S-phase cells, was added for 30 min after EdU labeling (Supplementary Figure S4F). Induction of pCHK1 S317 and presence of CYCLIN A could only be detected in the S-phase population (Supplementary Figure S4F), confirming that the populations were pure. These results suggest increased ATR signaling can also occur in the absence of replication following depletion of PNUTS.

### ATR signaling does not correlate with DNA damage or RPA loading after depletion of PNUTS

ATR is also well known to be activated by DNA double strand breaks, such as caused by IR (48). We therefore next compared PNUTS-depleted cells with IR-treated control siRNA transfected cells to address whether the high ATR activity after PNUTS depletion could correlate with DNA-damage. Higher levels of DNA damage markers pATM S1981, pDNAPK S2056, pCHK2 T68 and γH2AX, but lower levels of pCHK1 S317, were observed in IR-treated control cells (1 and 6 h after 10 Gy) compared to PNUTS-depleted cells (Figure 3D,E). Furthermore, the lack of DNA-damage signaling in PNUTS-depleted cells was not caused by a reduced ability to activate ATM or DNAPK, as this occurred normally after IR (Supplementary Figure S4G). The high ATR activity in PNUTS-depleted cells is therefore not likely caused by DNA damage.

RPA-ssDNA is a primary signal for ATR activation (e.g. reviewed in (4)), and can be assessed by measuring the amount of RPA loaded onto chromatin. We therefore compared the levels of RPA loading in non-treated cells tansfected with PNUTS siRNA and IR-treated cells transfected with control siRNA. Although pCHK1 S317 was higher in non-treated PNUTS-depleted cells compared to IR-treated control siRNA transfected cells 6 h after 10 Gy, RPA loading was lower (Figure 3F and G compared to 3D). This suggested a lack of correlation between ATR signaling and RPA loading after depletion of PNUTS. To further explore this, we co-depleted PNUTS and RPA70, an essential component of the RPA complex (reviewed in (49)). Remarkably, in cells co-depleted for PNUTS and RPA70 ATR-dependent pCHK1 S345 was as high as in cells depleted for PNUTS alone (Supplementary Figure S5A,B). High pCHK1 S345 was dependent on depletion of PNUTS, as higher pCHK1 S345 was observed in cells depleted for PNUTS and RPA70 compared to cells depleted for only RPA70 (Supplementary Figure S5B). As expected, co-depletion of RPA70 with PNUTS strongly reduced pRPA S33 (Supplementary Figure S5A,B). The high pCHK1 S345 was not caused by residual chromatin-bound RPA in the RPA70 and PNUTS co-depleted cells, as these cells had reduced RPA chromatin loading, but similar amounts of pCHK1 S345 compared to cells depleted for PNUTS alone 6 h after 10 Gy (Supplementary Figure S5C). Therefore, although our results do not exclude a contribution, they clearly show that the high ATR signaling after depletion of PNUTS is not correlated with enhanced amounts of RPA-ssDNA.

### R-loops are formed after depletion of PNUTS but likely play a minor role in the high ATR signaling

**Figure 4.**
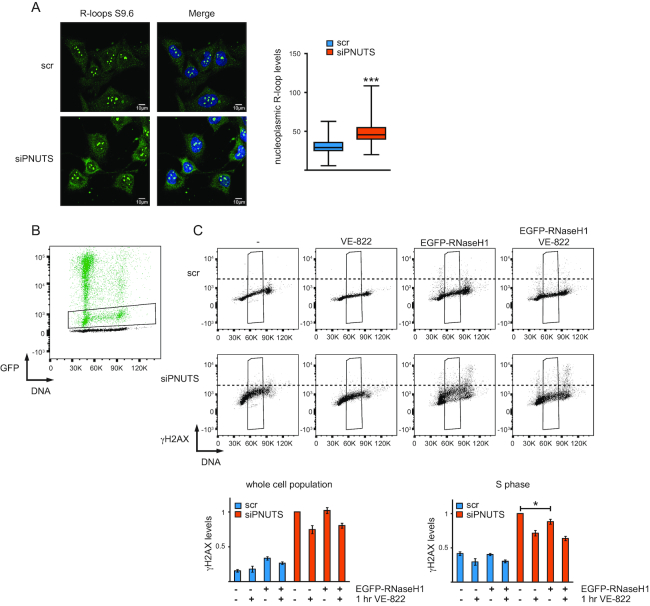
Depletion of PNUTS promotes R-loops, but overexpression of EGFP-RNaseH1 has only minor effects on ATR signaling. (**A**) Immunofluorescence analysis of R-loops in PNUTS depleted and control siRNA transfected cells at 72 h after siRNA transfection. The intensity of the nucleoplasmic staining is plotted. At least 50 cells from three independent experiments were scored. ***P < 0.001, by the Mann–Whitney test. (**B**) Representative flow cytometry chart showing GFP intensity versus DNA content. PNUTS depleted and control siRNA transfected cells were transiently transfected with EGFP-RNaseH1 at 24 h after siRNA transfection, and harvested at 72 h after siRNA transfection. Chart shows overlay of EGFP-RNaseH1 transfected (green) and non-EGFP-RNaseH1 transfected cells (black). Cells with moderate levels of EGFP-RNaseH1 expression were selected as indicated (black box). (**C**) Flow cytometry chart showing γH2AX staining versus DNA content in PNUTS depleted or control siRNA transfected cells with and without VE-822 for 1 h and with and without transient EGFP-RNaseH1 overexpression (selected for moderate levels of GFP expression as shown in B). Samples treated with the same siRNA oligonucleotides, were barcoded with pacific blue and mixed prior to staining as in 3E). Quantifications show relative, median γH2AX levels in the whole cell population or in the selected S-phase cells (*n* = 3). Error bars represent SEM. *P < 0.05 using two-tailed Student's *t*-test. Note that VE-822 reduces γH2AX in S-phase less than in Figure 3A, this is likely due to differences in the fixation protocol (required to preserve GFP intensity), which prolonged incubation time after wash-out of VE-822.

As R-loops recently have been proposed to play a role in ATR activation (50), we next addressed whether they might play a role in the increased ATR signaling after depletion of PNUTS. Interestingly, increased amounts of R-loops could be observed in cells transfected with PNUTS siRNA compared to cells transfected with control siRNA both by immunofluorescence and dot blotting using the S9.6 antibody (Figure 4A and Supplementary Figure S5D). Moreover, moderate levels of EGFP-RNaseH1 overexpression caused a partial reduction in ATR-dependent γH2AX in S-phase in cells transfected with PNUTS siRNA, but not in cells transfected with control siRNA (Figure 4B,C). However, in the whole cell population γH2AX levels were similar (Figure 4C), and upon higher levels of EGFP-RNaseH1 overexpression, γH2AX levels increased in all phases both in PNUTS siRNA and control siRNA transfected cells (data not shown). R-loops may therefore contribute to, but are not likely to be the major underlying cause, of the high ATR signaling after depletion of PNUTS.

### High ATR signaling does not strictly require common ATR activators after depletion of PNUTS

**Figure 5.**
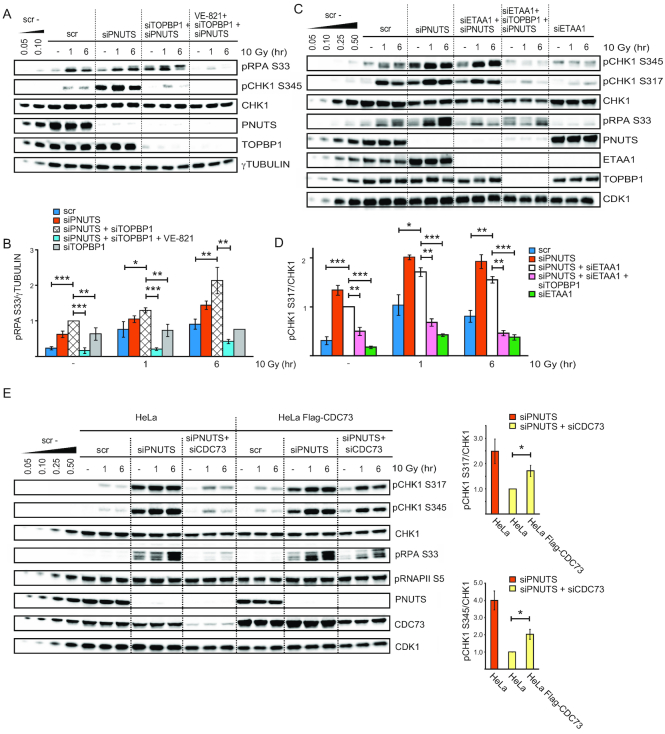
CDC73, but not TOPBP1 nor ETAA1, is required for high ATR-dependent phosphorylation of both CHK1 and RPA after PNUTS depletion. (**A** and **B**) Western blot and quantifications (*n* = 3) from cells transfected with scr, siPNUTS, and siRNA against TOPBP1 (siTOPBP1) harvested at 72 h after siRNA transfection and 1 and 6 h after 10 Gy. VE-821 was added 30 min prior to 10 Gy. For the siTOPBP1 10 Gy 6 h sample error bar was emitted in the quantifications as experiment was performed two times. Western blot for siTOPBP1 alone is shown in Supplementary Figure S5E. (**C** and **D**) Western blot and quantifications (*n* = 3) from cells transfected with scr, siPNUTS, siTOPBP1 and siRNA against ETAA1 (siETAA1) harvested at 48 h after siRNA transfection and 1 and 6 h after 10 Gy. (**E**) Western blot analysis and quantifications of scr, siPNUTS or CDC73 siRNA (siCDC73) transfected HeLa cells or HeLa cells stably expressing siRNA-resistant Flag-CDC73 treated with IR (10Gy) as indicated. Bar charts show quantification of pCHK1 S345 and pCHK1 S317 versus CHK1 levels at 6 h after 10 Gy (*n* = 3). Error bars indicate SEM and statistical significance was calculated by the two-tailed Student's two sample *t*-test. *P < 0.05, **P < 0.01, ***P < 0.001

We further addressed the involvement of other known key upstream ATR activating proteins, namely TOPBP1 and ETAA1. Though pCHK1 S345 was reduced, ATR-dependent pRPA S33 was not reduced in cells co-depleted for TOPBP1 and PNUTS compared to cells depleted for PNUTS alone, in the absence or presence of IR (Figure 5A and B). Thus, in PNUTS depleted cells TOPBP1 is required for the high ATR-mediated phosphorylation of CHK1 S345, but not of RPA S33. Notably, transfection of TOPBP1 siRNA alone did not greatly alter pRPA S33 (Figure 5B and Supplementary Figure S5E), confirming that the enhanced pRPA S33 in cells co-depleted for PNUTS and TOPBP1 was dependent on PNUTS depletion. Conversely, upon co-depletion of PNUTS with ETAA1, pRPA S33 was reduced, but pCHK1 S345/S317 was not greatly altered, compared to cells depleted for PNUTS alone (Figure 5C and D). Again the enhanced pCHK1 S317/S345 was dependent on PNUTS depletion, as pCHKS317/S345 was much lower in cells depleted for ETAA1 alone compared to cells transfected with PNUTS siRNA (Figure 5C and D). Triple depletion of PNUTS, ETAA1 and TOPBP1 suppressed both pCHK1 S317/S345 and pRPA S33 (Figure 5C and D). Together, these results are in agreement with recent findings suggesting that TOPBP1 is required for pCHK1 S317/S345 and ETAA1 for pRPA S33 (45,51). We conclude that neither TOPBP1 nor ETAA1 appear to be required for PNUTS-dependent ATR activity in general, but rather play essential downstream roles in the phosphorylations of specific substrates such as CHK1 and RPA, respectively.

To further characterize known ATR regulators following depletion of PNUTS, we closely compared their levels in cells transfected with PNUTS or control siRNA 24 or 48 h after siRNA transfection. Levels of ATR and ATRIP were not detectably altered (Supplementary Figure S5F). However, we found that ETAA1 was increased in PNUTS-depleted cells compared to cells transfected with control siRNA, particularly at 48 h after siRNA transfection (Supplementary Figure S5F). Upon close examination, CLASPIN and TOPBP1 were also slightly increased at 48 h (Supplementary Figure S5F). The co-depletions of PNUTS with ETAA1 or TOPBP1 nevertheless suggest that the ATR signaling can occur independently of either of these factors, though they are required for downstream phosphorylations (Figure 5A–D). Also, after IR, CLASPIN levels were downregulated, but pCHK1 S317 was higher in PNUTS-depleted cells relative to cells transfected with control siRNA (Supplementary Figure S6A), suggesting CLASPIN is not essential for enhanced ATR signaling upon PNUTS downregulation. The increased levels of ETAA1, CLASPIN and TOPBP1 are thus not likely the cause behind the high ATR signaling after depletion of PNUTS. However, their upregulation may be a consequence as ATR was recently shown to promote the transcription and protein stability of certain factors (52).

### pRNAPII-CTD interacting protein CDC73 is required for the high ATR signaling and the G2 checkpoint after depletion of PNUTS

**Figure 6.**
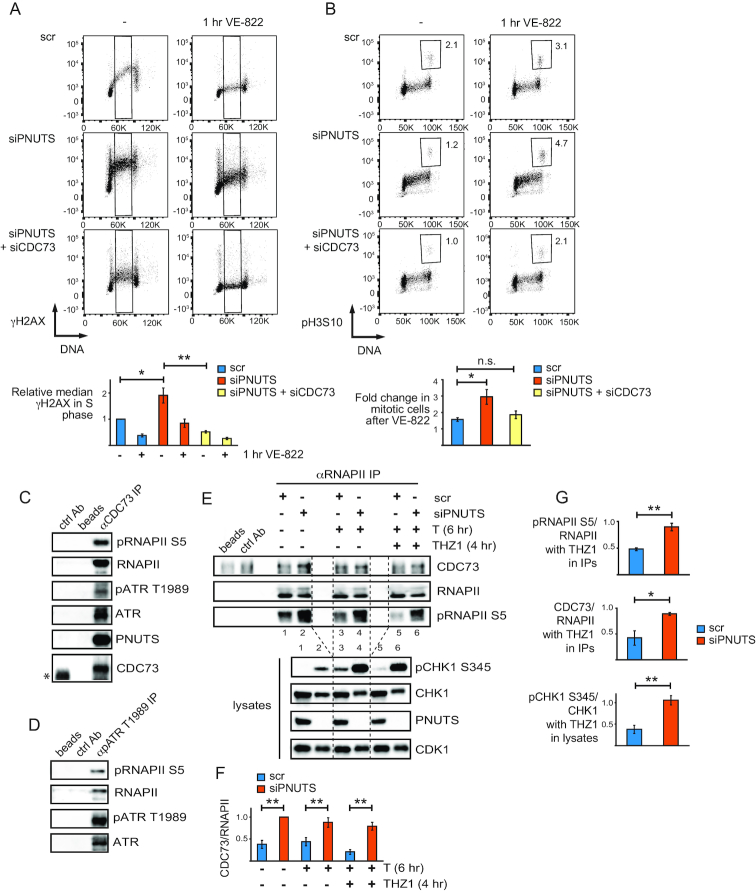
CDC73 is required for high ATR signaling in S-phase and activation of the endogenous G2 checkpoint after PNUTS depletion, and interacts with ATR and RNAPII. (**A**) Flow cytometry charts showing γH2AX staining versus DNA content as in 3A) of scr, siPNUTS or siPNUTS and siCDC73 transfected cells harvested at 72 h after siRNA transfection with and without 1 h treatment with VE-822. Quantifications show relative median γH2AX levels in indicated S-phase cells (black box). (*n* = 3) *P < 0.05, **P < 0.001 based on two-tailed two sample Student's *t*-test. (**B**) Flow cytometry charts showing phosphohistone H3 Ser 10 (pH3S10) staining versus DNA content of cells treated as in 6A). Mitotic cells were selected based on DNA content and high pH3S10 staining as indicated. Numbers indicate percentages of mitotic cells. Quantifications show fold increase in mitotic cells after 1h VE-822 for each siRNA condition. *P < 0.05, based on two-tailed two sample Student's *t*-test. (**C**) Western blot analysis of immunoprecipitations from HeLa cell lysates, using a control antibody (ctrl Ab), no antibody (beads) or anti-CDC73 antibodies (αCDC73 IP). *Indicates IgG band from the control antibody, which migrated slightly faster than CDC73 in the western blot. (**D**) Western blot analysis of immunoprecipitations as in C), but using anti-pATR T1989 antibodies (αpATR T1989 IP). (**E**) Western blot analysis of immunoprecipitations as in (C), but using RNAPII antibodies recognizing both the phosphorylated and the non-phosphorylated RNAPII (αRNAPII IP). Immunoprecipitations were performed on lysates from scr and siPNUTS transfected cells harvested at 72 h after siRNA transfection, with and without thymidine for 6 h and THZ1 for 4 h. Upper western blot shows immunoprecipitations, and lower blot shows corresponding lysates. (**F**) Bar chart showing quantifications from three independent experiments performed such as E, of CDC73 relative to RNAPII in western blots from RNAPII immunoprecipitations. (**G**) Bar charts showing fold changes of THZ1 and thymidine treated samples relative to samples treated with thymidine alone for respective siRNA oligonucleotides from quantifications of western blots from three independent experiments performed such as (E). pRNAPII S5 relative to RNAPII and CDC73 relative to RNAPII values were from the immunoprecipitations, and pCHK1 S345/CHK1 values were from the corresponding lysates. For quantifications of CDC73 from immunoprecipitations, background (value of band in beads alone), was substracted during the quantifications. *P < 0.05, **P < 0.01 based on the two-tailed Student's two sample *t*-test. Error bars represent SEM.

Our results showing a connection between RNAPII CTD phosphorylation and ATR signaling (Figure 2B,C and Supplementary Figure S3) suggest that the CTD may be acting as a signaling platform for ATR activity. We therefore searched for factors that might participate in signaling from phosphorylated RNAPII CTD towards ATR. In the literature, we identified three proteins, BRCA1, PRP19 and CDC73, that associate with hyperphosphorylated RNAPII and have been linked to ATR (53–58). We found that co-depletion of BRCA1 or PRP19 with PNUTS did not reduce the high ATR signaling (data not shown). However, co-depletion of CDC73 with PNUTS reduced both pCHK1 S317/S345 and pRPA S33, but not pRNAPII S5, in the presence or absence of IR (Figure 5E). The reduction in pCHK1 S345 phosphorylation after co-depletion was observed with several siRNA oligonucleotides against CDC73 (four out of five) (Supplementary Figure S6B). Furthermore, expression of siRNA resistant Flag-CDC73 partially rescued the effects on pCHK1 S317/S345 and pRPAS33 downregulation after co-depletion of CDC73 with PNUTS (Figure 5E), excluding siRNA off-target effects. The reduction in ATR signaling after co-depletion of CDC73 with PNUTS was not due to indirect cell cycle effects, because γH2AX in individual S-phase cells was significantly reduced under these conditions compared to cells depleted for PNUTS alone (Figure 6A and Supplementary Figure S6E).

We previously found that depletion of PNUTS activates an endogenous G2 checkpoint in unperturbed cells (23). As the G2 checkpoint depends upon ATR and its downstream target CHK1 (59), and co-depletion of CDC73 suppressed ATR signaling after depletion of PNUTS (Figures 5E and 6A), we addressed whether co-depletion of CDC73 might also suppress activation of the endogenous G2 checkpoint. For this purpose, we measured entry into mitosis after addition of VE-822 to siRNA-transfected cells. In agreement with our previous results using caffeine and a CHK1 inhibitor (23), after addition of VE-822, more cells transfected with PNUTS siRNA entered into mitosis compared to cells transfected with control siRNA (Figure 6B). Remarkably, co-transfection of CDC73 siRNA suppressed this effect (Figure 6B). Notably, to ensure that only entry into mitosis from cells arrested in G2 phase was being assessed, we added VE822 for only 1 h, a time point well below the average duration of G2, which is ∼3 h in HeLa cells (60), and we also only counted cells with a *4C* DNA content (Figure 6B).

We next addressed whether co-depletion of CDC73 might also influence R-loops, which we found to be increased after depletion of PNUTS (Figure 4A and Supplementary Figure S5D). Interestingly, we found that the levels of R-loops were reduced after co-depletion with CDC73 compared to cells treated with PNUTS siRNA alone (Supplementary Figure S6C). As CDC73 plays a role in transcription, this supports our hypothesis that the enhanced levels of R-loops after depletion of PNUTS are also caused by effects on transcription. Altogether, these results suggest that CDC73 plays an important role in ATR activation that is counteracted by PNUTS, and are consistent with a role for CDC73 in signaling from phosphorylated RNAPII CTD to ATR.

CDC73 interacts genetically with the ATR homologue Mec1 in *Saccharomyces cerevisiae*, and a physical interaction has been proposed but not previously shown (57). Furthermore, RNAPII is a known interacting partner of CDC73 (61,62), and in *S. cerevisae* it was shown that CDC73 binds the RNAPII CTD in a phosphorylation-dependent manner (62). To examine CDC73, ATR and RNAPII interactions in HeLa cells, we performed co-immunoprecipitation (co-IP) experiments of endogenous proteins. Indeed, co-IPs using a CDC73 antibody pulled down RNAPII, pRNAPII S5, ATR and pATR T1989 (Figure 6C). As pATR T1989 is thought to be an autophosphorylation site (63), this indicates that catalytically active ATR associates with CDC73. Interestingly, PNUTS and PP1 were also detected in the CDC73 co-IPs (Figure 6C and Supplementary Figure S6D). We verified that the immunoprecipitations were specific by using lysates from cells depleted of CDC73, which pulled down less ATR and RNAPII (Supplementary Figure S6D). Furthermore, the depletion of CDC73 was only partial and significant amounts of CDC73 were present in the co-IPs from cells transfected with CDC73 siRNA (Supplementary Figure S6D, CDC73-high exposure), which may explain the residual ATR and RNAPII pulled down under these conditions. Next, we performed ATR co-IPs to address whether ATR and pRNAPII S5 could physically associate. To enrich for active ATR in these experiments, we used pATR T1989 antibodies. This efficiently pulled down ATR and faint bands corresponding to pRNAPII S5 and RNAPII could also be detected, suggesting an interaction in live cells (Figure 6D). Moreover, to address whether hyperphosphorylation of the RNAPII CTD after depletion of PNUTS might promote binding to CDC73, we performed RNAPII co-IPs using an antibody that recognizes both the phosphorylated and non-phosphorylated forms of RNAPII. Indeed, more CDC73 was pulled down in RNAPII immunoprecipitates after depletion of PNUTS compared to control siRNA transfected cells (Figure 6E). In these experiments we also induced replication stress with thymidine and added THZ1. In line with our results showing that THZ1 reduced RNAPII CTD phosphorylation and ATR signaling in control-, but not in PNUTS-depleted cells (Figure 2C), immunoprecipitated RNAPII was less phosphorylated and less CDC73 was pulled down in the control-, but not in the PNUTS-depleted cells after THZ1 treatment (Figure 6E, F lanes 3 versus 5 and 4 versus 6 and Figure 6G). Of note, in these experiments we measured pRNAPII S5, but other CTD-phosphorylation sites, such as S2 or S7 may play a role but are not shown here. Also, all the co-IPs were performed after treatment with the endonuclease benzonase, strongly suggesting that the interactions were not mediated by DNA. Altogether these results suggest that CDC73, ATR and RNAPII may interact in live cells, and that CDC73 interacts with the RNAPII CTD in a phosphorylation-dependent manner also in humans. These results thus strongly support a role for phosphorylated RNAPII and CDC73 in the high ATR activity after PNUTS depletion.

## DISCUSSION

**Figure 7.**
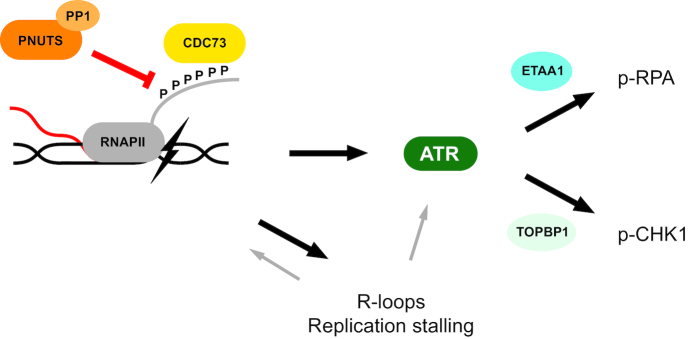
Model for regulation of ATR signaling via PNUTS-PP1, the phosphorylated CTD of RNAPII and CDC73. We envision that stalling of elongating RNAPII, caused by DNA damage or other obstacles (e.g. reviewed in (8)), causes hyperphosphorylation of the RNAPII CTD (see main text for details) which increases its binding to CDC73. Once bound to the RNAPII CTD, CDC73 either directly or indirectly activates ATR. PNUTS-PP1 suppresses ATR activity by dephosphorylating the RNAPII CTD, thus reducing the binding of CDC73 to RNAPII CTD and activation of ATR. R-loops formed under these conditions may also contribute to ATR signaling, but are likely to play a minor role. Furthermore, during S-phase the stalled RNAPII, R loops and ATR activity likely also cause replication stalling, which may further contribute to induce ATR signaling through canonical activation or potentially via further increasing RNAPII stalling in a positive feedback loop. Our results also indicate that TOPBP1 and ETAA1 can direct the ATR activity towards pCHK1 S317/345 and pRPA S33, respectively. Altogether our model is consistent with the model originally proposed by the groups of Sancar and Ljungman, where RNAPII signals the presence of DNA damage by stalling as it encounters an obstacle during transcription elongation (6,7).

ATR kinase plays a central role in signaling after DNA damage and replication stress. Here, we show for the first time that the RNAPII phosphatase PNUTS-PP1 suppresses ATR signaling. Furthermore, we have identified a well-known RNAPII binding protein, CDC73, as a novel factor mediating ATR activation via the RNAPII CTD and being required for the high ATR signaling in PNUTS-depleted cells. Our results suggest that ATR signaling is restrained by PNUTS-PP1 mediated dephosphorylation of RNAPII CTD, and thus support a role for RNAPII in ATR signaling. Moreover, our results support recent findings that TOPBP1 and ETAA1 may direct ATR activity towards different substrates. Altogether, based on these results we propose a new model for ATR activation via CDC73, RNAPII and PNUTS-PP1 (Figure 7).

Interestingly, this model is in line with previous reports showing that perturbation of transcription can induce ATR activation in the absence of DNA damage and prior to detection of replication-stress (7,64). We envision that signaling to ATR by pRNAPII CTD via CDC73 may be a general event that occurs upon RNAPII stalling, regardless of context. As Mec1 was shown to promote removal of RNAPII at sites of transcription-replication conflict (57), viewed in light of our results, ATR activity might thus promote removal of stalled RNAPII also outside of S-phase. This is likely important, because stalled RNAPII could create an obstacle for further transcription in a region which might e.g. contain an essential- or tumor suppressor gene. In agreement with prolonged RNAPII stalling being detrimental to the cell, it has been shown to be a strong signal for apoptosis (65).

In addition to the high ATR signaling, depletion of PNUTS also caused an accumulation of cells in S-phase and decreased EdU uptake (Supplementary Figure S4A,B,E), indicating increased replication stalling. These effects might be expected as stalled RNAPII and R-loops after PNUTS depletion may create obstacles for the replication fork (reviewed in (66)), and the high ATR activity likely also contributes to slowing down replication (reviewed in (44)). Nevertheless, our results strongly suggest that the high ATR activity after depletion of PNUTS cannot simply be caused by canonical signaling via enhanced replication stress. First of all, it was also observed in the absence of replication in G1-phase after PNUTS depletion (Figure 3B, C) and was higher than expected compared to ATR signaling induced by HU-generated replication stress (Supplementary Figure S4B–E). In addition, the high ATR activity did not correlate with RPA-ssDNA (Figure 3D, F, G and Supplementary Figure S5A–C), which is considered to be the main signal for replication stress-induced ATR activity (44). Furthermore, suggesting that it is rather RNAPII phosphorylation which is important for the high ATR signaling after depletion of PNUTS, short-term incubation with the CDK7-inhibitor THZ1 reduced both RNAPII phosphorylation and ATR signaling in control siRNA but not in PNUTS siRNA transfected cells (Figure 2C and Supplementary Figure S3B,C). Moreover, RNAPII and CDC73 may be directly involved in ATR signaling as they were found to interact with ATR (Figure 6C, D). Phosphorylation of the RNAPII CTD was also important for the interaction between CDC73 and RNAPII (Figure 6E–G), and co-depletion of CDC73 with PNUTS strongly reduced ATR signaling (Figures 5E and 6A). Altogether, our results thus point to a signaling pathway involving ATR, RNAPII and CDC73 which is continuously counteracted by PNUTS-PP1. On the other hand, in S-phase, canonical signaling from stalled replication forks may also contribute to promoting ATR activation after depletion of PNUTS (see model in Figure 7). Still, it is tempting to speculate that replication stalling after depletion of PNUTS may further enhance RNAPII stalling and thus create a positive feedback loop by increasing RNAPII/CDC73-mediated ATR activity (see model in Figure 7).

Interestingly, R-loops were enhanced after depletion of PNUTS and suppressed by co-depletion of CDC73 (Figure 4A and Supplementary Figure S5D). However, EGFP-RNaseH1 only partially suppressed ATR-dependent γH2AX in S-phase cells transfected with PNUTS siRNA (Figure 4C), suggesting that R-loops may contribute to the high ATR activity but likely play a minor role. As R-loops were recently shown to cause ATR activation at centromeres in mitosis by a mechanism proposed to involve RPA-ssDNA (50), one speculation could be that depletion of PNUTS causes small amounts of ssDNA-RPA associated with R-loops, and that the resulting structure may confer some specificity which enhances ATR signaling. On the other hand, there is an intimate connection between stalled RNAPII and R-loops (67). It was recently shown that overexpression of RNaseH1 can cause release of stalled RNAPII, suggesting that R-loops can promote RNAPII stalling (32). Therefore, another possibility might be that R-loops might contribute to ATR signaling by leading to stalling of RNAPII and subsequent RNAPII CTD phosphorylation.

We found that RNAPII CTD phosphorylation was required for, but did not strictly correlate with, ATR signaling (e.g. Supplementary Figure S3D—compare lanes 1 and 2, pCHK1 S317 versus pRNAPII S5). However, RNAPII CTD phosphorylation is a frequent event during the normal transcription cycle. The most studied phosphorylation sites are S5 and S2, and in brief, studies have shown that phosphorylation on S5 is high at the start of the gene and thereafter gradually decreases, while inversely, phosphorylation on S2 increases throughout the gene (Reviewed in (37,68)). The widespread presence of S2 and S5 RNAPII CTD phosphorylation implies that a strict linear correlation with ATR activation is unlikely, as it would suggest that ATR becomes activated merely as a consequence of normal transcription. Thus, it is likely that only a subpopulation of pRNAPII CTD is responsible for signaling to ATR. Supporting this, only stalling of the elongating form of RNAPII caused increased ATR signaling (7). As elongation is associated with phosphorylation on S2 and phosphorylation on S5 is enhanced upon RNAPII stalling, e.g. at sites of UV damage or at splice sites located at gene-internal regions (14,69), one conceivable mechanism is that dual S2 and S5 phosphorylation might be required for signaling to ATR. Supporting this, CDC73 bound more tightly to dually- than to singly-phosphorylated pRNAPII CTD in vitro (62). Nevertheless, the situation is likely to be more complex, as the human CTD contains 52 heptapeptide repeats and different modifications, and combinations of these, exist (68).

Of note, in the alternative splicing response to UV, pRNAPII CTD was proposed to occur downstream of ATR activation, and ATR activation to occur independently of transcription in HaCaT cells (70). These results may appear to be contradictory to ours. However, we did not detect any reduction in pRNAPII S5 after ATR inhibition during replication stress in HeLa cells (Supplementary Figure S2C) suggesting ATR is not always upstream of RNAPII CTD phosphorylation. Furthermore, the differing results may be explained by the existence of several pathways for ATR activation acting in parallel, e.g. via RNAPII, via ssDNA-RPA, and via unknown pathways. The contribution from each pathway is likely to vary between cell types and with different stresses.

Our results point to a new role for CDC73 in ATR activation. CDC73 is a component of the PAF1 complex, including PAF1, CTR9, LEO1, RTF1 and WDR61, involved in all stages in RNAPII transcription (61). However, CDC73 does not appear to be essential for transcription as its depletion in HeLa cells was found to both up and down-regulate mRNA expression (71). In *S. cerevisae* CDC73 was found to act downstream of Mec1 in collisions of transcription and replication (57). Our results suggest CDC73 in association with RNAPII can also act upstream of ATR activation. Interestingly, CDC73 is also a well known tumor suppressor gene. It is currently not clear how CDC73 acts as a tumor suppressor, though roles in Wnt signaling, regulation of P53 and CYCLIN D levels and homologous recombination repair have been suggested (72–75). ATR activity protects genome integrity by stabilizing stalled forks during replication stress and promoting DNA repair and checkpoint activation (76). In addition, ATR activity can promote apoptosis in non-cycling cells, which implies the majority of cells in humans (77). Therefore, CDC73 could potentially protect against cancer by promoting RNAPII-mediated ATR activity, leading to cell death in non-cycling cells with DNA damage. Consistent with this interpretation, PNUTS, which counteracts CDC73 in ATR activation, is a putative proto-oncogene (78).

In conclusion, this work sheds light upon a previously proposed pathway for ATR activation via the RNAPII machinery. We have identified novel factors involved, including CDC73, the phosphorylated CTD of RNAPII and PNUTS-PP1. Future studies are likely to uncover more details into this understudied and highly relevant pathway for ATR activation.

## Supplementary Material

Supplementary Data
