# Supplementary material for: Regulation of ATR activity via the RNA polymerase II associated factors CDC73 and PNUTS-PP1
Source: Nucleic Acids Res. 2018 Dec 12;47(4):1797–813. doi: 10.1093/nar/gky1233 (PMC6393312; doi:10.1093/nar/gky1233)
Supplement: Supplementary Data [file gky1233_supplemental_files.pdf]

SUPPLEMENTARY DATA:

Supplementary figure legends:

Figure S1. A) Western blot analysis and quantifications of scr and siPNUTS transfected U2OS cells at 2 or 6 hr after addition of thymidine. Bar chart under the western blot shows results from the same experiment. Bar charts to the right show quantification from 5 independent experiments of pCHK1 S317 relative to CHK1 or pRPA S33 relative to γTUBULIN or CDK1. **B)** Western blot analysis and quantifications (n=3) of scr and siPNUTS transfected HeLa cells at 1 or 6 hr after 10 Gy. VE-821 was added 30 min prior to IR. **C)** Western blot analysis and quantifications (n=3) of siPNUTS HeLa cells at 2 or 6 hr after thymidine. VE-821 was added 30 min prior to thymidine. For A-C) Error bars indicate SEM and statistical significance was calculated by the two-tailed students two sample t-test. *p<0.05, **p<0.01, ***p<0.001. **D)** scr, siPNUTS cells or cells transfected with siRNA against NIPP1 (siNIPP1) at 1 or 6 hr after 10 Gy. Bar chart under the western blot shows results from the same experiment. Experiment was performed two times with similar results.

Figure S2. A) Western blot analysis and quantifications from experiment as in 1D, 6 hr after thymidine. Bar chart shows quantification of pCHK1 S317 relative to CHK1 (n=3). Error bars indicate standard error of the mean (SEM) and statistical significance was calculated by the two-tailed students two sample t-test. ***p<0.001 **B)** Western blot analysis and quantifications from experiment performed as in 1D). Bar chart below shows quantification from the same experiment. **C)** Western blot analysis of scr or siPNUTS transfected cells without or with thymidine (16hr T). VE-822 was added for 2, 5, 15, 30, or 60 min to indicated samples 16hr after addition of T. Charts show fold changes after VE-822 treatement at various timepoints vs T 16 hr alone, for respective

siRNA oligos from quantifications from western blot of pCHK1 S317/CHK1, pCHK1 S345/CHK1 and pRPA S33/CDK1. Experiment was performed two times with similar results.

Figure S3. A) Western blot analysis and quantifications (n=3) of HeLa cells 72 hr after siRNA transfection with scr or siRNA against SSU72 (siSSU72) with or without 2 or 6 hr thymidine. **B)** Western blot analysis and quantifications (n=3) from experiments performed as in 2C). The charts to the right show fold changes for THZ1 and thymidine samples 4 hr after THZ1, relative to the 2 hr thymidine sample for the respective siRNA oligos for pRNAPII S2 or pRNAPII S7 relative to CDK1. **C)** Western blot analysis of scr or siPNUTS transfected cells with or without IR (10 Gy, 1 or 2 hr). THZ1 or cycloheximide was added 10 min after IR to the indicated samples. Samples with or without THZ1/CHX were collected together to allow direct comparison. The right charts show fold changes of THZ1 + 10 Gy relative to 10 Gy alone at the same timepoints after IR for the respective siRNA oligonucleotides for pRNAPII S5 relative to CDK1 and pCHK1 S317/S345 relative to CHK1 levels (n=3). For pCHK1 S345 and pCHK1 S317, statistical significance was calculated using the two-tailed one sample students t-test, testing whether the fold change after THZ1 for the respective siRNA oligonucleotides was different from one. Notably, using this test, no statistical significance could be detected after THZ1 treatment for neither pCHK1 S317 nor S345 for the siPNUTS samples. For pRNAPII S5, statistical significance was calculated using the two-tailed two sample students t-test, comparing fold change after THZ1 between siPNUTS and scr transfected samples. *$p<0.05$, **$p<0.01$. **D)** Representative western blot and resulting quantification from HeLa cells with or without transcriptional inhibitors THZ1, DRB, triptolide or translational inhibitor cycloheximide. Inhibitors were added to cells 60 min prior to 10 Gy and samples were harvested at 15, 30 and 60

min. CDC25A levels verify effects of cycloheximide on a short-lived protein. Quantifications beneath western blot shows results from the same experiment. The experiment was performed three times under resembling conditions with similar results.

Figure S4. A) Histograms from the experiment in 3A, showing cell cycle profiles based on DNA content. **B)** Flow cytometry charts showing EdU incorporation versus DNA content of HeLa cells with or without 80 µM HU for 24 hr and siPNUTS transfected cells 48 hr after siRNA transfection. **C)** Western blot from same experiment as in B). **D)** Bar charts show quantifications from western blots from three independent experiments such as B). Statistical significance was tested using the two-tailed two sample t-test, ***p<0.001, *p<0.05. **E)** Bar charts show quantifications from flow cytometry analysis from three independent experiments such as B). Statistical significance was tested using the two-tailed two sample t-test, but siPNUTS transfected cells were not found to be significantly different from 80 µM HU samples when EdU incorporation or percentage cells in S phase was compared. **F)** Western blot and quantifications from experiment as in 3B. Cells were labeled with EdU for 1 hr, followed by 30 min incubation with thymidine, harvested and sorted. **G)** Western blot analysis and quantifications of scr or siPNUTS transfected cells without or with IR (10 Gy, 1 or 6 hr)). (n=7 for pATM S1981, and n=5 for pDNAPK S2056).

Figure S5 A) and B) Representative western blots of cells transfected with scr, siPNUTS, and siRNA against RPA70 (siRPA70) harvested at 72 hr after siRNA transfection and 1 and 6 hr after 10 Gy. VE-821 was added 30 min prior to 10 Gy. (n=3). **C)** Immunofluorescence analysis and quantifications (n=3) of pre-extracted cells treated as in A) and B). Quantifications of pCHK1 S345/CHK1 are from western blots of samples treated in parallel with the immunoflourescence samples. Statistical significance was tested using the two-tailed two sample t-test**,** **p<0.01 **D)** Dot blots

from scr or siPNUTS transfected cells harvested at 72 hr after siRNA transfection showing R-loops (n=5). **E)** Western blots of cells transfected with scr, siPNUTS, and siTOPBP1 harvested at 72 hr after siRNA transfection and 1 and 6 hr after 10 Gy. **F)** Western blot analysis of scr or siPNUTS transfected cells at 24 and 48 hr after siRNA transfection.

Figure S6 A) Western blot analysis of scr or siPNUTS transfected cells at 72 hr after siRNA transfection, without and with IR (10 Gy, 6h). **B)** Western blot and quantifications from representative experiment of cells transfected with scr, siPNUTS, and five different oligonucleotides against CDC73, siCDC73 #1, #2, #3, #4 and #5 harvested 48 hr after siRNA transfection. (n=3). siCDC73 #2 is also called siCDC73. **C)** Immunofluorescence analysis of R-loops in cells transfected with scr, siPNUTS and scr or siPNUTS and siCDC73 at 72 hr after siRNA transfection. The intensity of the nucleoplasmic staining is plotted. At least 50 cells from three independent experiments were scored. Statistical significance was determined using the Mann-Whitney test. **D)** Western blot from immunoprecipitation experiment performed as in 6C on lysates from cells transfected with scr or siCDC73 #1, at 72 hr after siRNA transfection. **E)** Histograms from the experiment in 6A) showing cell cycle profiles based on DNA content.

Supplementary Materials and Methods:

Dot Blot

U2-OS cells were transfected with control, PNUTS, harvested after 72 hours and lysed in lysis buffer (100 mM NaCl, 10 mM Tris pH 8.0, 25 mM EDTA pH 8.0, 0.5% SDS) with 140 µg/ml Proteinase K, at 37ºC overnight. Nucleic acids were extracted using standard phenol-chloroform extraction and re-suspended in DNase/RNase-free water. Nucleic acids were fragmented using a restriction enzymes cocktail, containing 20U of each of the following restriction enzymes: EcoRI (FD0274, Thermo Scientific), BamHI (FD0054, Thermo Scientific), HindIII (FD0504, Thermo Scientific), Bsp1407I (FD0933, Thermo Scientific) and XhoI (FD0694, Thermo Scientific). Then, half of each sample was digested with 40U of RNaseH enzyme (MB08501, NZYTech) to serve as negative control, for about 48 hours at 37ºC. Digested nucleic acids were purified with phenol-chloroform extraction, re-suspended in DNase/RNase-free water and quantified. DNA samples were diluted to a concentration of 10 µg/mL and 100 µL of solution (1 µg DNA) was loaded per well, into a positively charged nylon membrane (RPN203B, GE Healthcare), using the Bio-Dot® Microfiltration System (1703938, Bio-Rad). The DNA was cross-linked through UV irradiation (UV Stratalinker 2400, Stratagene) and membranes were blocked for 1 hr at room temperature with 5% milk in PBS 1x containing 0,05% Tween 20. Incubation with the primary antibodies (anti-dsDNA (sc-58749, Santa Cruz) and S9.6 (ENH001, Kerafast)) was performed at 4ºC overnight, followed by incubation with secondary antibody (anti-Mouse-HRP (170-6516, Biorad)) for 1 hour at room temperature. Detection was achieved using enhanced chemiluminescence substrates (RPN2209, GE Healthcare).

Figure S1
Landsverk et al.

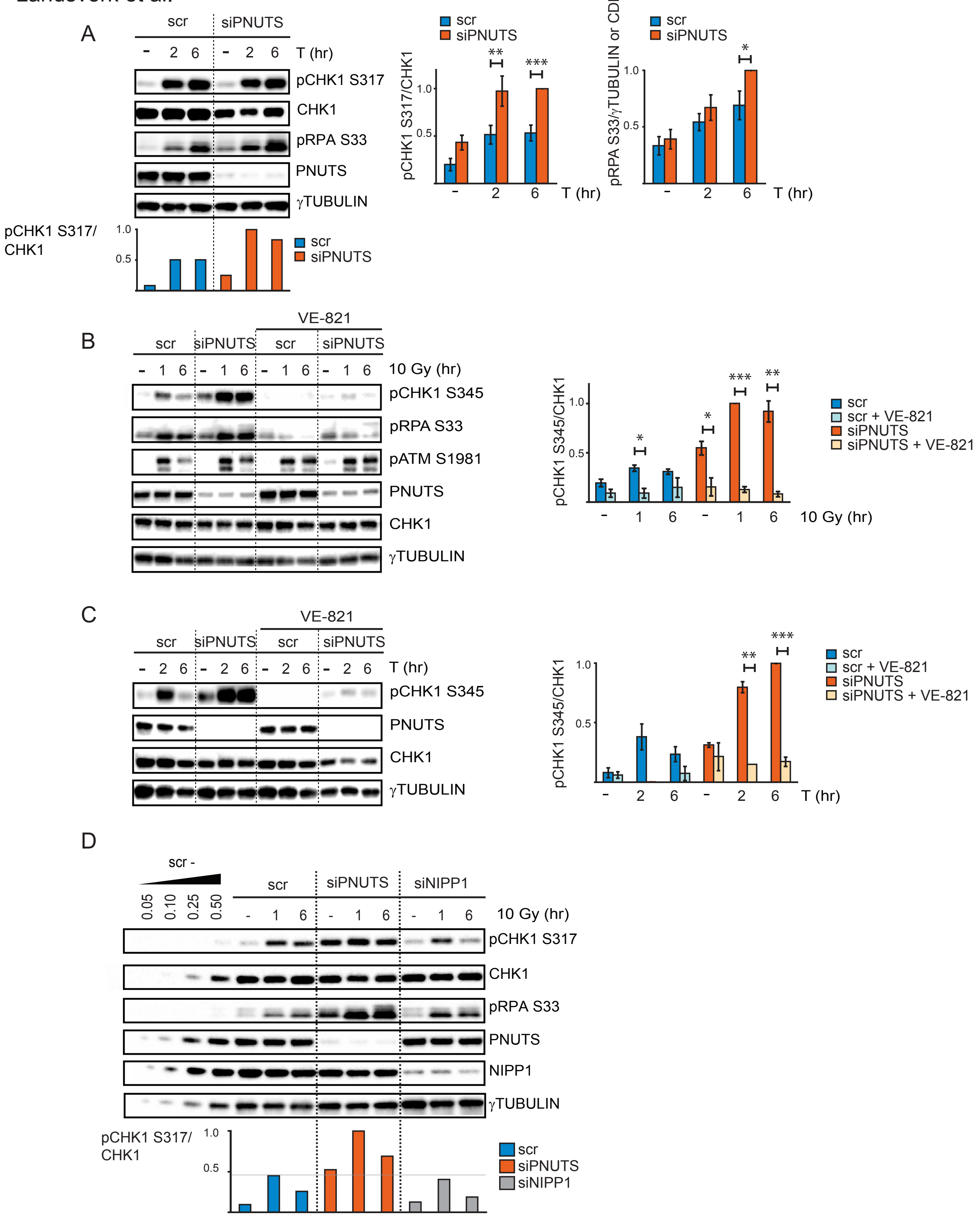

Figure S2
Landsverk et al.

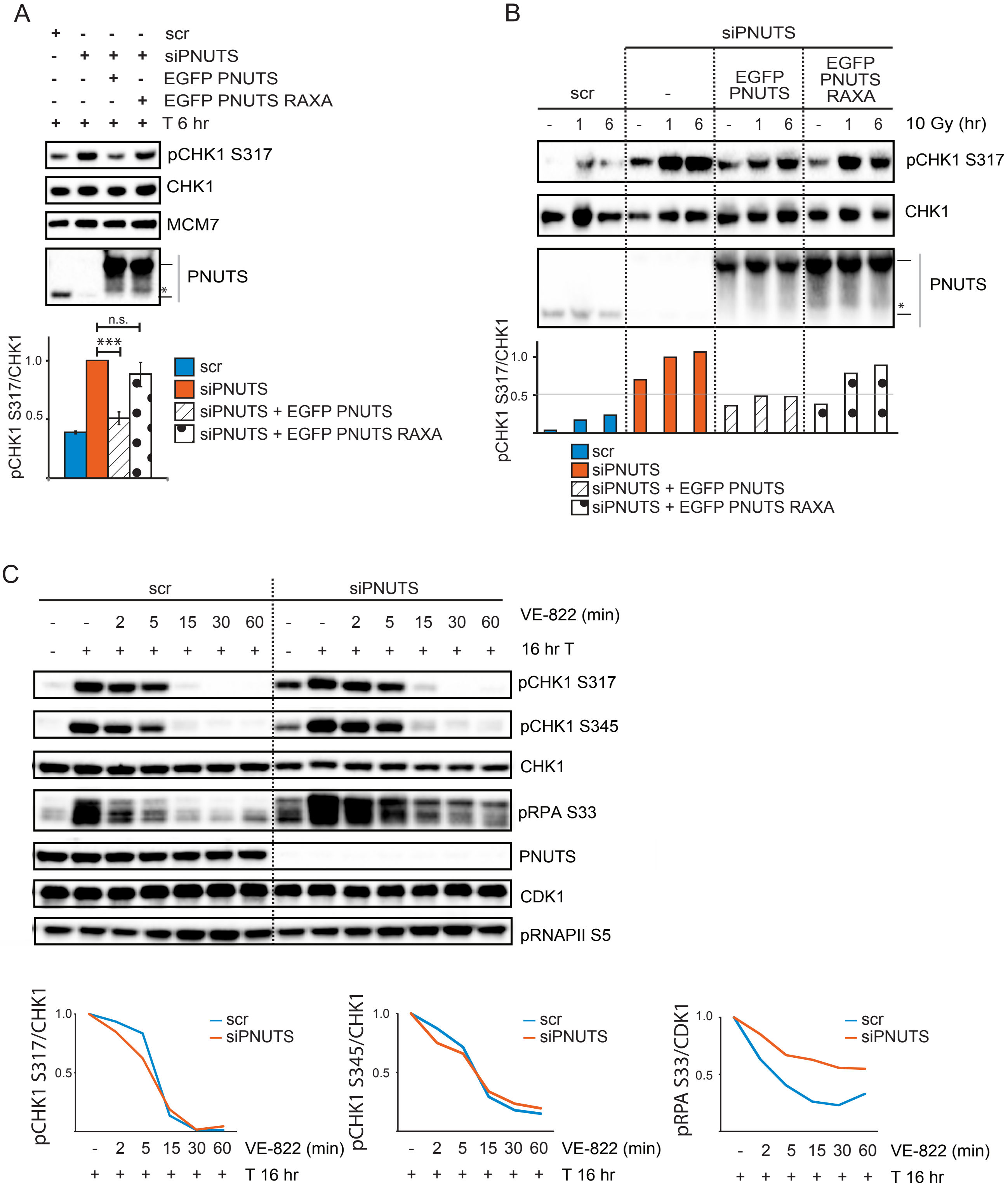

Figure S3
Landsverk et al.

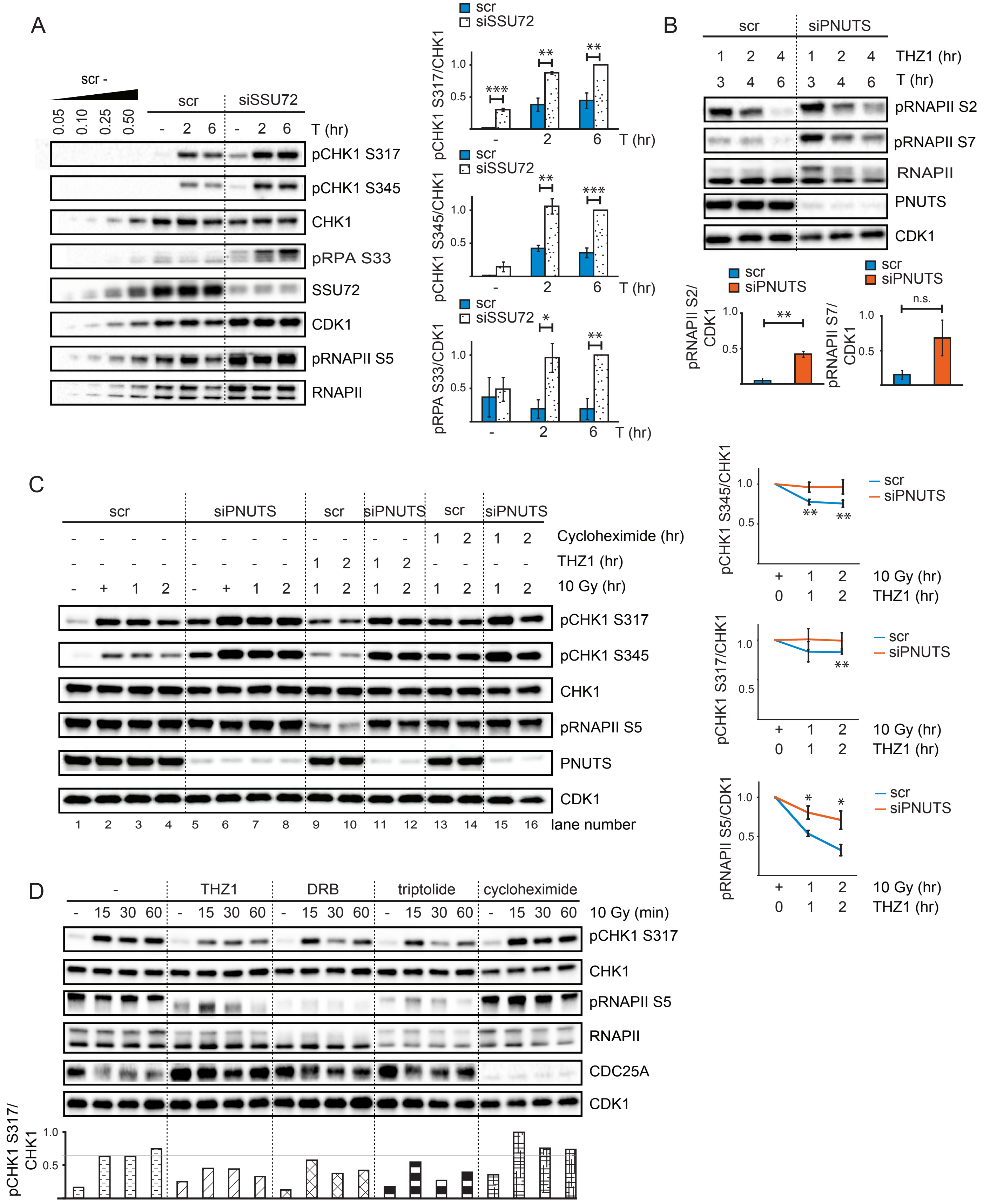

Figure S4
Landsverk et al.

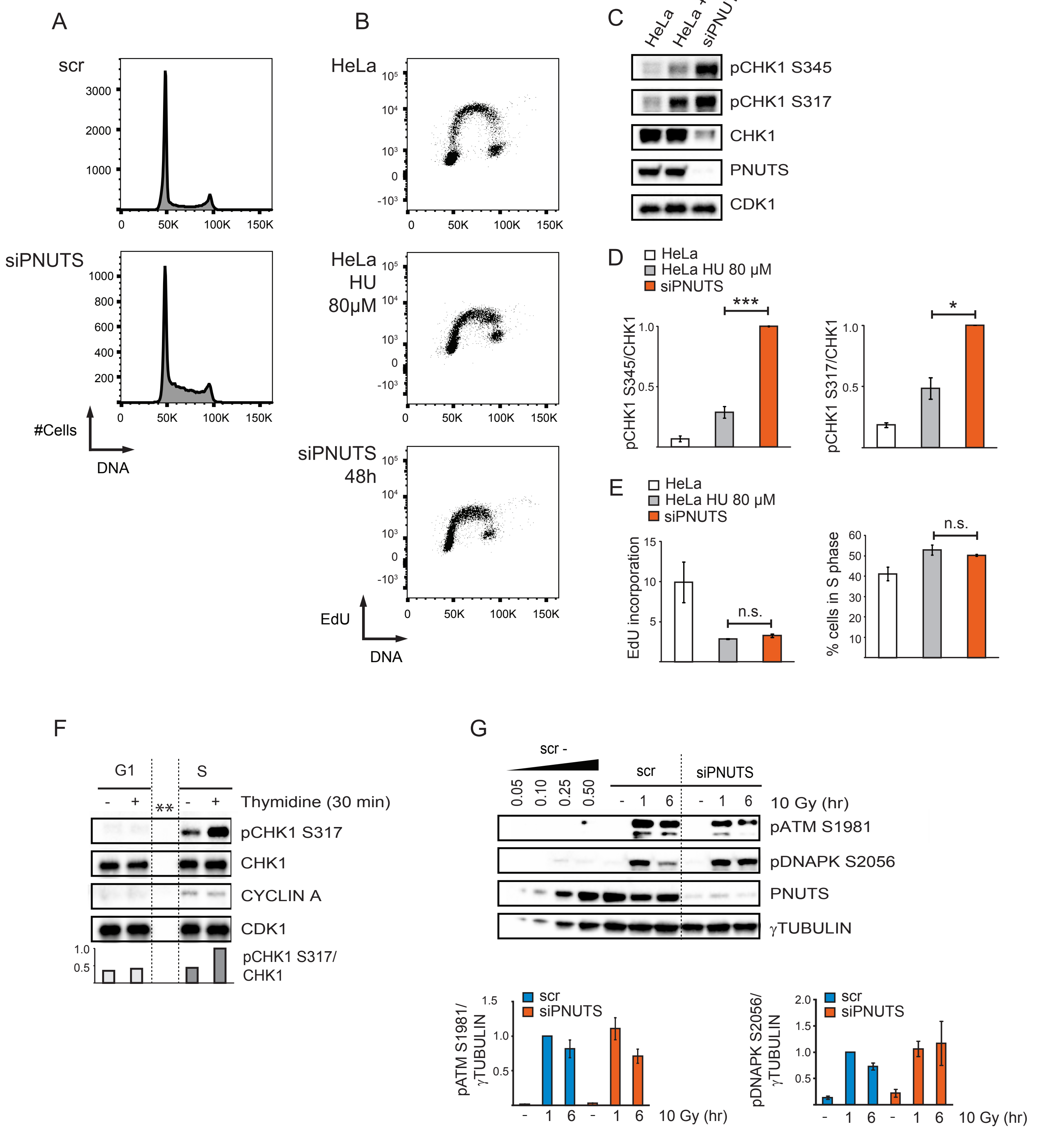

Figure S5
Landsverk et al.

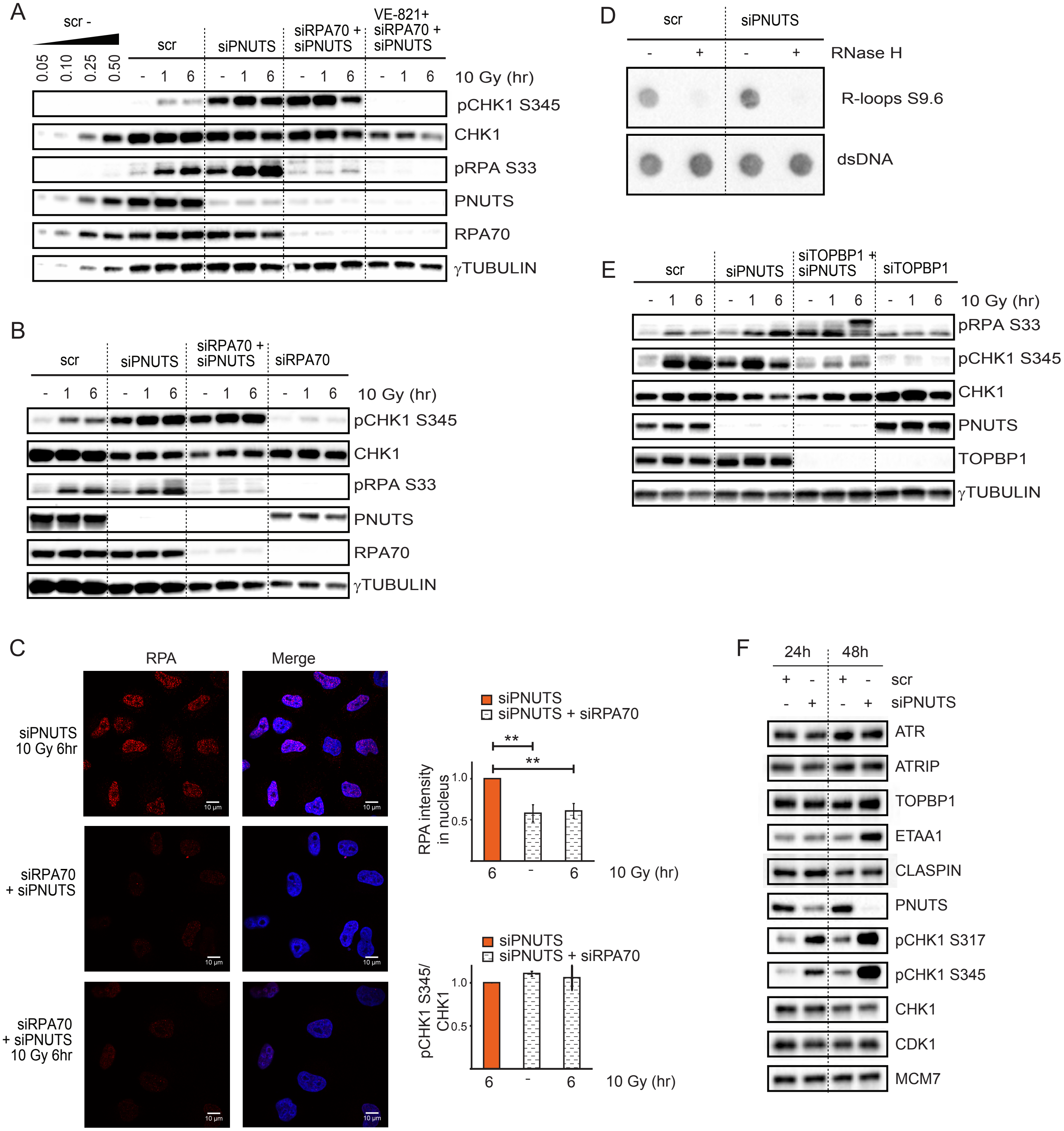

Figure S6
Landsverk et al.

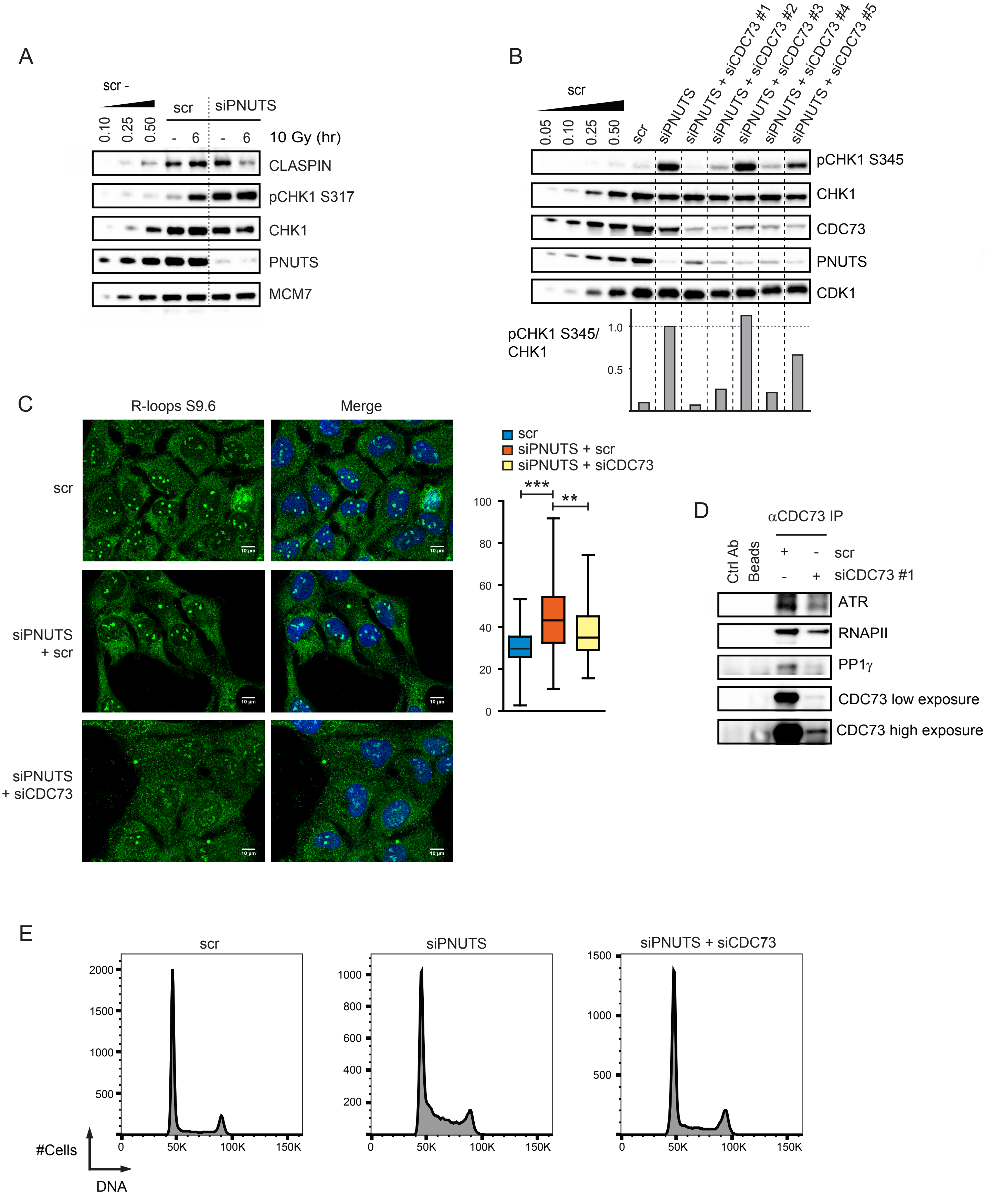

Table S1. siRNA oligonucleotide sequences

Name of siRNA	Sequence	Manufacturer	Reference
Scr (scrambled control siRNA of siPNUTS #1)	GGUUUCUGUCAAAUGCAAACGGCUU	Thermo Fisher (stealth siRNA)	(1)
siPNUTS #1	GGUGGUUUCUGACAAGUACAACCUU	Thermo Fisher (stealth siRNA)	(1)
siPNUTS #2 (also called siPNUTS)	GCAAUAGUCAGGAGCGAUA (silencer select s328)	Thermo Fisher	
siCDC73 #1	AAGCGUCAACAUCGGCAAGUA	Sigma-Aldrich	(2)
siCDC73 #2 (also called siCDC73)	AAACAAGGUUGUCAACGAGAA	Sigma-Aldrich	(2)
siCDC73 #3	CUGAACAGAUUAGGUCUUU (SASI_Hs01_00126024)	Sigma-Aldrich	
siCDC73 #4	GGAUCUCGAACACCCAUUA	Sigma-Aldrich	(3)
siCDC73 #5	CUAUCAAGACUGAUCUAGA	Sigma-Aldrich	(3)
siRPA70	GGACAAGUUCUUUCCUCUUAUUG	Sigma-Aldrich	(4)
siTOPBP1	AGACCUUAAUGUAUCAGUA	Sigma-Aldrich	(5)
siNIPP1	GGAACCUCACAAGCCUCAGCAAAUU	Thermo Fisher (stealth siRNA)	(6)
siETAA1	GAGCAAAACAAGAGGAAUU	Sigma-Aldrich	(7)
siSSU72	GGAGCUUCCUGUUGUUCAU (SASI_Hs01_00024012)	Sigma-Aldrich	

1. Landsverk HB, *et al.* (2010) The protein phosphatase 1 regulator PNUTS is a new component of the DNA damage response. *EMBO reports* 11(11):868-875.
2. Hahn MA, *et al.* (2012) The tumor suppressor CDC73 interacts with the ring finger proteins RNF20 and RNF40 and is required for the maintenance of histone 2B monoubiquitination. *Human molecular genetics* 21(3):559-568.
3. Herr P, *et al.* (2015) A genome-wide IR-induced RAD51 foci RNAi screen identifies CDC73 involved in chromatin remodeling for DNA repair. *Cell discovery* 1:15034.
4. Liu S, *et al.* (2011) ATR autophosphorylation as a molecular switch for checkpoint activation. *Molecular cell* 43(2):192-202.
5. Kousholt AN, *et al.* (2012) CtIP-dependent DNA resection is required for DNA damage checkpoint maintenance but not initiation. *The Journal of cell biology* 197(7):869-876.
6. Minnebo N, *et al.* (2013) NIPP1 maintains EZH2 phosphorylation and promoter occupancy at proliferation-related target genes. *Nucleic acids research* 41(2):842-854.
7. Haahr P, *et al.* (2016) Activation of the ATR kinase by the RPA-binding protein ETAA1. *Nature cell biology* 18(11):1196-1207.

Table S2. Antibodies used

Antibody target	Manufacturer	Reference	Use
PNUTS	BD Biosciences		WB
phosphoCHK1 Ser317	Cell Signaling Technology		WB
phosphoCHK1 Ser345	Cell Signaling Technology		WB
phosphoATM Ser1981	Cell Signaling Technology		WB
phosphoCHK2 Thr68	Cell Signaling Technology		WB
ATR	Cell Signaling Technology		WB
CHK1		(1)	WB
γTUBULIN (GTU-88)	Sigma		WB
CDK1 (sc-54)	Santa Cruz Biotechnology		WB
RNAPII (F-12)	Santa Cruz Biotechnology		WB
MCM7 (DCS-141)	Santa Cruz Biotechnology		WB
phospho RPA32 Ser33	Bethyl		WB
phosphoDNAPK S2056	Abcam		WB
phosphoRNAPII S5 (3E8)	Millipore		WB
phosphoRNAPII S7 (4E12)	Millipore		WB
phosphoRNAPII S2 (3E10)	Millipore		WB
CDC73	Bethyl		WB, IP
phosphoATR Thr1989	GeneTex		WB, IP
ETAA1		(2)	WB
R-loops (S9.6, ENH001)	Kerafast		IF, Dotblot
NIPP1 (sc-393991)	Santa Cruz Biotechnology		WB
TOPBP1	Abcam		WB
CLASPIN	Cell Signaling		WB
ATRIP	Millipore		WB
γH2AX	Abcam		FC
phosphoHISTONE H3 S10	Millipore		FC
CDC25A (DCS-120)	Santa Cruz Biotechnology		WB
PP1γ (sc-6108)	Santa Cruz Biotechnology		WB
CYCLIN A (sc-751)	Santa Cruz Biotechnology		WB
RPA70	Cell Signaling Technology		WB, FC
Double stranded DNA (sc-58749)	Santa Cruz Biotechnology		Dotblot
RPA32 (MABE286)	Millipore		IF
SSU72 (D3I2D)	Cell Signaling		WB

WB: Western blotting, FC: flow cytometry, IF: immunofluorescence microscopy

Peroxidase-conjugated secondary antibodies were from Jackson Immunoresearch.

Alexa-conjugated secondary antibodes were from Thermo Fisher.

1. Sorensen CS*, et al.* (2003) Chk1 regulates the S phase checkpoint by coupling the physiological turnover and ionizing radiation-induced accelerated proteolysis of Cdc25A. *Cancer Cell* 3(3):247-258.
2. Haahr P*, et al.* (2016) Activation of the ATR kinase by the RPA-binding protein ETAA1. *Nature cell biology* 18(11):1196-1207.
